# Silencing of Doublecortin-Like (DCL) Results in Decreased Mitochondrial Activity and Delayed Neuroblastoma Tumor Growth

**DOI:** 10.1371/journal.pone.0075752

**Published:** 2013-09-26

**Authors:** Carla S. Verissimo, Rachel Elands, Sou Cheng, Dirk-Jan Saaltink, Judith P. ter Horst, Maria N. Alme, Chantal Pont, Bob van de Water, Bjarte Håvik, Carlos P. Fitzsimons, Erno Vreugdenhil

**Affiliations:** 1 Division of Medical Pharmacology, Leiden/Amsterdam Center for Drug Research, Leiden University Medical Center, Leiden, the Netherlands; 2 Prosensa Therapeutics B.V., Leiden, the Netherlands; 3 Department of Biomedicine, K. G. Jebsen Centre for Research on Neuropsychiatric Disorders, University of Bergen, Bergen, Norway; 4 Division of Toxicology, Leiden/Amsterdam Center for Drug Research, Leiden University Medical Center, Leiden, the Netherlands; 5 Dr. E. Martens Research Group for Biological Psychiatry, Department of Clinical Medicine, University of Bergen, Bergen, Norway; 6 Center for Medical Genetics and Molecular Medicine, Haukeland University Hospital, Bergen, Norway; 7 Centre for Neuroscience, Swammerdam Institute for Life Sciences, University of Amsterdam, Amsterdam, the Netherlands; 8 Department of Human Genetics, Migraine Research Group, Leiden University Medical Center, Leiden, the Netherlands; University of Texas Health Science Center at San Antonio, United States of America

## Abstract

Doublecortin-like (DCL) is a microtubule-binding protein crucial for neuroblastoma (NB) cell proliferation. We have investigated whether the anti-proliferative effect of DCL knockdown is linked to reduced mitochondrial activity. We found a delay in tumor development after DCL knockdown *in vivo* in doxycycline-inducible NB tumor xenografts. To understand the mechanisms underlying this tumor growth retardation we performed a series of *in vitro* experiments in NB cell lines. DCL colocalizes with mitochondria, interacts with the mitochondrial outer membrane protein OMP25/ SYNJ2BP and DCL knockdown results in decreased expression of genes involved in oxidative phosphorylation. Moreover, DCL knockdown decreases cytochrome c oxidase activity and ATP synthesis. We identified the C-terminal Serine/Proline-rich domain and the second microtubule-binding area as crucial DCL domains for the regulation of cytochrome c oxidase activity and ATP synthesis. Furthermore, DCL knockdown causes a significant reduction in the proliferation rate of NB cells under an energetic challenge induced by low glucose availability. Together with our previous studies, our results corroborate DCL as a key player in NB tumor growth in which DCL controls not only mitotic spindle formation and the stabilization of the microtubule cytoskeleton, but also regulates mitochondrial activity and energy availability, which makes DCL a promising molecular target for NB therapy.

## Introduction

Neuroblastoma (NB) is the most commonly diagnosed cancer in infants [1] and the most frequent solid extracranial neoplasm in children under five years of age [2,3]. NB origins from neural crest cells, which are the precursors of the sympathetic nervous system [4]. With the currently available therapies the survival rate of patients with advance stage NB remains below 50% [5]. Great efforts have been done towards a more effective and less toxic therapy for NB. These efforts include the identification of novel molecular targets that play a crucial role in NB tumorigenic processes, such as proliferation, alternative energy metabolism, relative (acquired) resistance to apoptosis, angiogenesis and/or metastasis [6,7,8].

We have previously proposed the Doublecortin-like kinase (*DCLK1*) gene as an attractive molecular target for NB therapy [7,9]. DCLK-derived proteins belong to doublecortin (DCX) family and include the microtubule associated proteins (MAPs) DCLK-long and doublecortin-like (DCL). DCL and DCLK-long are highly expressed in neuroblasts and are vital for neuroblast proliferation, migration and differentiation [10,11]. Silencing of DCLK-derived MAPs results in cell-cycle arrest and apoptosis in NB cells [9,11].

Structurally, DCL exhibits high amino acid sequence identity with DCX in both the two microtubule-binding domains (DCX-domains) and their C-terminal serine/proline (S/P)-rich domain [12]. Because of their DCX-domains, DCLK-derived MAPs play a role in microtubule stabilization and mitotic spindle formation [10,11]. In addition, aided by protein-protein interactions via its S/P-rich domain, DCL interacts with several proteins and is involved in the intracellular transport of glucocorticoid receptors in neuronal progenitor cells and NB cells [13,14].

We have shown that DCL is highly expressed in human NB and glioma tumors [9]. Moreover, we found a high correlation between DCL expression and the expression of genes related to mitochondrial activity in human NBs. Surprisingly, gene expression profiling after DCLK-derived MAPs knockdown in NB cells indicated that mitochondria were among the most affected cellular components. Moreover, oxidative phosphorylation (OXPHOS) and oxidative stress were among the most affected biological process, suggesting an additional role for DCL in the regulation of the energy metabolism [9].

Mitotic stimuli result in the activation of mitochondrial bioenergetics by transcriptional activation of mitochondrial genes and induction of intracellular signaling pathways [15,16]. Cell proliferation is associated with increased rates of mitochondrial OXPHOS and glycolysis [17]. Both processes generate energy in the form of adenosine triphosphate (ATP) and biochemical intermediates for *de novo* biosynthesis of macromolecules [17,18]. These energetic pathways have been proposed as potential therapeutic targets for cancer therapy [19,20,21,22].

In this study, we investigate the effect of DCL knockdown on tumor growth in an *in vivo* NB xenograft model as well as on mitochondrial activity in NB cells. Our data show a key role of DCL in NB tumor development and in energy supply of NB cells, a function that has not been reported for the *DCLK1* gene or any other member of the DCX family.

## Materials and Methods

### Reagents and antibodies

The mouse monoclonal antibody against α-tubulin was purchased from Sigma-Aldrich Chemie B.V (Zwijndrecht, The Netherlands) and the secondary antibody anti-mouse-HRP from Tube-bio B.V. (Bevelandseweg, The Netherlands). A recently developed primary rabbit antibody targeting the DCL-specific sequence QRDLYRPLSSDDLDSVG-C was used [23]. Alexa Fluor® 488 goat anti-rabbit IgG, Alexa Fluor® 488 donkey anti-rabbit IgG and Alexa Fluor® 594 goat anti-mouse IgG were from Molecular Probes (Leiden, The Netherlands). The anti-rabbit-HRP antibody was from Santa Cruz (Tebu-Bio, Heerhugowaard, The Netherlands). 3-

(4,5-dimethylthiazol-2-yl)-2,5-diphenyltetrazolium bromide (MTT) was obtained from Sigma-Aldrich Chemie BV (Zwijndrecht, The Netherlands). 5 mg/milliliter MTT in phosphate-buffered saline (PBS, Life Technologies, Europe BV, Bleiswijk, The Netherlands) was freshly prepared before each determination. Doxycycline (Dox) and G418 were purchased from Sigma-Aldrich Chemie B.V (Zwijndrecht, The Netherlands). Dithiothreitol (DTT) was obtained from Sigma-Aldrich Chemie BV (Zwijndrecht, The Netherlands) and the Complete Protease Inhibitor Cocktail Tablets from Roche Diagnostics Nederland BV (Almere, The Netherlands).

### Cell culture and doxycycline treatment

Mouse N1E-115 neuroblastoma (NB) cells and African green monkey kidney COS-1 cells were cultured as previous described [9,11]. Doxycycline (Dox)-inducible NB stable cell lines were cultured in the presence of 500 µg/ml G418. The development of the Dox-inducible NB stable cell lines from N1E-115 was described by Verissimo et al. 2010 [9]. These cells (shDCL-2 or shDCL-3), in the presence of 1 µg/ml Dox, express a shRNA against DCL [9]. The shDCL-2 cell line expresses the shRNA with the sequence 5’-TCC-CGC-TGG-TCA-TCC-TGC-ATC-TTG-TTT-CAA-GAG-AAC-AAG-ATG-CAG-GAT-GAC-CAG-CTT-TTT-A-3’ and the complementary sequence is 5’-CGC-GTA-AAA-AGC-TGG-TCA-TCC-TGC-ATC-TTG-TTC-TCT-TGA-AAC-AAG-ATG-CAG-GAT-GAC-CAG-C-3’. The sequence of the shRNA expressed in shDCL-3 cell line is 5’-TCC-CGG-TCA-TCC-TGC-ATC-TTG-TTG-TTT-CAA-GAG-AAC-AAC-AAG-ATG-CAG-GAT-GAC-CTT-TTT-A-3’ and 5’-CGC-GTA-AAA-AGG-TCA-TCC-TGC-ATC-TTG-TTG-TTC-TCT-TGA-AAC-AAC-AAG-ATG-CAG-GAT-GAC-C-3’. In addition, we have developed a negative control (NC) NB Dox-inducible stable cell line from N1E-115 as described previously [9]. A scramble shRNA nucleotide sequence was cloned in pINV-7 vector (TaconicArtemis GmbH, Cologne, Germany) as previously described [24]. The NC stable cell line expresses a scramble shRNA with the sequence 5’-TCC-CGC-TGT-CGC-TCT-TTC-GAG-TTT-ATT-CAA-GAG-ATA-AAC-TCG-AAA-GAG-CGA-CAG-CTT-TTT-A-3’ and complementary sequence 5’-CGC-GTA-AAA-AGC-TGT-CGC-TCT-TTC-GAG-TTT-ATC-TCT-TGA-ATA-AAC-TCG-AAA-GAG-CGA-CAG-C-3’. The Dox-inducible NB stable cells were treated with 1 µg/ml Dox or vehicle (Veh, milli-Q water) for 72 hours. We used high glucose (4.5 g/L) DMEM medium to keep the Dox-inducible NB cells in culture. For the challenge assay, low (1 g/L) glucose DMEM medium (Life Technologies, Europe BV, Bleiswijk, The Netherlands) was used.

### Transfection

For recovering DCL expression in Dox-inducible NB stable cells that were in the presence of 1 µg/ml Dox for 72 hours, we transfected these cells with 125 ng pcDNA3.1 vector (Invitrogen, Breda, The Netherlands) containing the nucleotide sequence for DCL full-length [11] or pcDNA3.1 empty plasmid using lipofectamine 2000 (Invitrogen, Breda, The Netherlands) following the manufacturer’s instructions. In addition, the medium with Dox was replaced by medium with vehicle (milli-Q water). Forty-eight hours after transfection, cell proliferation was investigated or the cells were harvested for western blot, cytochrome c activity or ATP synthesis assays. Using TransIT®-COS Transfection kit (Mirus Bio LLC, Madison, WI), COS-1 cells were transfected with one of the different DCL truncations subcloned into pDsRed2-N1 vector as described previously [13]. The DCL truncations were generated from DCL-DsRed2-N1 by PCR [13]. The primers used are indicated in Table S1.

### RNA isolation and quantitative real-time PCR

Cells were harvested and the RNA was isolated using TRIzol reagent (Invitrogen, Breda, The Netherlands) according to the manufacturer’s specifications. The RNA concentration and purity were determined using a NanoDrop spectrophotometer (NanoDrop products, Wilmington, DE). Quantitative real-time PCR (RT-qPCR) was performed using ABI Prism 7900HT Sequence Detection system (Applied Biosystems, Foster City, CA) according to the manufacturer’s instructions. Messenger RNA expression of Cox7c (Mm01545088_g1), Cox6a2 (Mm00438295_g1), Ndufa1 (Mm00444593_m1), Ndufa13 (Mm00445751_m1), Ncl (Mm00834059_g1), Nenf (Mm00840262_m1), Pttg1 (Mm00479224_m1) and S100a6 (Mm00771682_g1) was analyzed using a target-specific assay (Assays-on-demand Gene expression system; Applied Biosystems). The expression levels were normalized to 18S rRNA (Hs99999901_s1, Applied Biosystems). shRNA expression was detected using Applied Biosystems custom TaqMan® small RNA Assay and following the manufacturer’s instructions (Applied Biosystems, Foster City, CA). The target sequence for shDCL-2 was 5’-ACAAGAUGCAGGAUGACCAGC-3’, for shDCL-3 was 5’-ACAACAAGAUGCAGGAUGACC-3’ and for scramble shRNA (NC) was 5’-AUAAACUCGAAAGAGCGACAGC-3’. The expression levels were normalized to *snoRNA202* (Applied Biosystems).

### Western blot analysis

Protein lysates, SDS-PAGE, and western blotting were performed as described by Vreugdenhil et al [11]. The expression of DCL was normalized to α-tubulin. Analysis and quantification of the relative optical densities were performed using ImageJ software [25].

### Cell proliferation assay

Cell proliferation and survival were determined using the colorimetric 3-(4,5-dimethylthiazol-2-yl)-2,5-diphenyltetrazolium bromide (MTT) assay as described previously [26], with some modifications. In brief, N1E-115 cell lines were seeded in 96-well culture plates at a seeding density of 3000 cells/well. 72 hours after treating the cells with 1 µg/ml Dox or Veh, 0.83 mg/ml MTT solution was added, and the cells were incubated for 4 hours at 37°C, 5% CO_2_. Afterwards, the medium with MTT was removed and 100 µl of DMSO was added to each well for solubilization of formazan crystals. The optical density was measured at 540 nm with a reference wavelength of 630 nm, using a FLUOstar Optima plate reader (BMG LABTECH GmbH, Offenburg, Germany).

### Ethics statement and xenograft tumors assay

Animal experiments were approved by the Local Committee for Animal Health, Ethics and Research of Leiden University and conducted in accordance with the European Communities Council Directive 86/609/EEC. Eight-week-old female BALB/c athymic nude mice (Charles River Laboratories, Cologne, Germany), five mice per group, were injected subcutaneously into the right flank with 1 x 10^6^ cells (one of the Dox-inducible NB stable cell lines) using 25-G needles. Cells were resuspended in 100 µl PBS. Three days following cell inoculation, Dox-diet, 200 mg/kg doxycycline (Plexx BV, Elst, The Netherlands) was provided or the mice continued receiving the control diet (Plexx BV, Elst, The Netherlands). Tumor growth was monitored by caliper and mice were weighted every three days. Tumor volume was calculated as previously described [27]. At day 14, animals were euthanized and tumors were extracted.

### Immunohistochemistry

Tumor tissue was formalin fixed and paraffin embedded. Four micrometer sections were stained with H&E or subjected to immunohistochemistry. Sections were deparaffinized, rehydrated through an alcohol gradient to water, and subjected to heat-induced antigen retrieval in 0.01 M citrate buffer pH 6.0 for 10 minutes. Endogenous peroxide activity was blocked with 1.5% (v/v) peroxide in methanol for 30 minutes at room temperature and nonspecific binding was blocked with 2% (v/v) normal horse serum (Vector Labs) or 2% (w/v) bovine serum albumin (Sigma Aldrich) in PBS for 30 minutes. Sections were incubated overnight at 4°C with rabbit polyclonal antibody Ki67 (Novocastra Laboratories Ltd, Newcastle, United Kingdon), with cleaved caspase-3 (Asp175) (5A1E) rabbit mAb (Cell Signaling Technologies, Leiden, The Netherlands) or with anti-α-tubulin mouse mAb (Sigma-Aldrich Chemie BV, Zwijndrecht, The Netherlands). The secondary antibodies Alexa Fluor® 488 donkey anti-rabbit and Alexa Fluor® 594 goat anti-mouse were incubated for one hour at room temperature. Tissue was counterstained with 1:10000 Hoechst for 5 minutes. Mouse N1E-115 cells were grown for 72 hours in cover slips coated with 200 ng/µl poly-L-lysine (Sigma-Aldrich Chemie B.V, Zwijndrecht, The Netherlands). Cells were incubated for 30 minutes at 37°C in DMEM medium containing 100 nM MitoTraker Orange CMTM Rosamine (Molecular Probes, Leiden, The Netherlands). Subsequently, N1E-115 cells were washed three times for five minutes with PBS-Tween 20 and fixed with methanol-EGTA (97:3). Next, 4% freshly prepared paraformaldehyde in PBS was added to the cells for 10 minutes. After three washes with PBS-Tween 20, cells were permeabilized and blocked with TBP (1% Triton-x-100, 1% bovine serum albumin (BSA) in PBS) for 1 hour at room temperature. Cells were incubated with the rabbit monoclonal anti-DCL antibody (1:500) for one hour. Following three times wash with PBS-Tween 20, Alexa Fluor® 488 goat anti-rabbit IgG (Life Technologies, Europe BV, Bleiswijk, The Netherlands) was added for one hour at room temperature. Nuclei were stained with 1:10000 Hoescht (Life Technologies, Europe BV, Bleiswijk, The Netherlands) for five minutes.

### Immunofluorescence and laser-scanning confocal microscopy

Immunofluorescence and laser-scanning microscopy in Dox-inducible NB stable cell lines from N1E-115 neuroblastoma cells and in African green monkey kidney COS-1 cells was performed as previously described [13]. and using Leica TCS SP8 confocal laser scanning microscope. COS-1 cells were transfected with different DCL fragments subcloned into pDsRed2-N1 vector as described previously [13]. EZ-Viewer software (Nikon Instruments, Europe BV, Amstelveen, The Netherlands), Leica application suite advanced fluorescence software (LAS AF, Leica Microsystems B.V., Rijswijk, The Netherlands) and ImageJ [25], were used for image analysis and protein co-localization studies. Quantification of colocalization score for DCL and mitochondria in stable NB cells was performed as described previously [13].

### Caspase-3 activity

Caspase-3 activity assay was performed as described previously [9].

### Yeast two-hybrid screen

The yeast 2-hybrid screen was performed with a Mouse Adult Brain_RP1 ULTImate Y2H^TM^ Library and the bait CARP/ANIA-4 (GI:5468526) cloned into pB27 (N-LexA-CARP-C) (Hybrigenics-Services, Paris, France). CARP/ANIA-4 is a 55 amino acid peptide that covers the 50 C-terminal amino acids of DCL [28]. 79.3 million possible interactions were screened and a proteome-wide interaction map (PIM) biological score (Global PBS) were used to classify interactions into confidence categories [29,30].

### Mitochondria isolation

Mitochondria were isolated as described previously [31] with some modifications. NB cells were harvested using trypsin (GIBCO, Invitrogen BV) and spun down at 1500 rpm for 5 minutes. Pellet was washed with DMEM medium and, subsequently with cold PBS. Cells were resuspended in 200 µl mitobuffer (20 mM HEPES pH 7.5, 250 mM sucrose, 1 mM EGTA, 1 mM EDTA, 10 mM KCl, 1.5 mM MgCl2, 1 mM DTT, protein inhibitor cocktail tablet (1 table per 10 ml mitobuffer) and nuclease free water) and lysed by sonication. The lysate were centrifuged at 3000 rpm for 20 minutes at 4°C and the supernatant was then spun down at 11000 rpm for 20 minutes at 4°C. The pellet that contains the mitochondria was washed once with mitobuffer and spun down at 11000 rpm. Subsequently, the pellet was resuspended in 200µl Enzyme Assay Buffer (Mitochondria Activity Assay kit, Bio-Connect BV, Huissen, The Netherlands) and stored at -80°C.

### Cytochrome C Oxidase Activity assay

The activity of cytochrome C Oxidase was measured in isolated mitochondria from Dox-inducible NB cells treated with 1 µg/ml Dox or vehicle (Veh, milli-Q water) for 72 hours. The measurements were performed in agreement with the advised procedure from Mitochondria Activity Assay (Cytochrome C Oxidase Activity assay) Kit (Bio-Connect BV, Huissen, The Netherlands). In short, we measured the reduction in absorption at the wavelength of 550 nm using UV/Vis UV-1700 PharmaSpec spectrophotometer (Shimadzu, Benelux BV, Hertogenbosch, The Netherlands) at different time points (5, 15, 25, 35 and 45 seconds). Samples were normalized to the amount of protein, which was determined using Pierce BCA protein assay (Thermo, Fisher Scientific, Landsmeer, The Netherlands). Furthermore, results were normalized for membrane integrity of the outer mitochondrial membrane (Cytochrome C Oxidase Activity Kit, Bio-Connect BV, Huissen, The Netherlands).

### ATP fluorescent assay

72 hours after 1 µg/ml Dox or Veh treatment, NB cells with inducible DCL knockdown were harvested and lysed using ATP Assay Buffer from the Colorimetric/Fluorometric Assay Kit (ITK Diagnostics BV, Uithoorn, The Netherlands). ATP levels were detected by a fluoremetric assay following the instructions described in Colorimetric/Fluorometric Assay (ITK Diagnostics BV, Uithoorn, The Netherlands) and using Viktor^2^ Multilabel plate reader. Results were normalized to the amount of protein that was determined performing Pierce BCA protein assay (Thermo, Fisher Scientific, Landsmeer, The Netherlands).

### Statistical analysis

Unless otherwise indicated, results presented are representative of three independent experiments run in triplicates. Student’s t-test and two-way ANOVA were carried out using GraphPad Prism 4.00 (GraphPad software, La Jolla, USA) and SPSS statistical software version 16.0 (SPSS Inc., Chicago, IL, USA). *P* values smaller than 0.05 were considered statistically significant. Results are expressed as mean ± standard error of the mean (s.e.m.).

## Results

### Validation of DCL silencing in Doxycycline (Dox)-inducible NB stable cell lines and effects on NB proliferation

**Figure 1:**
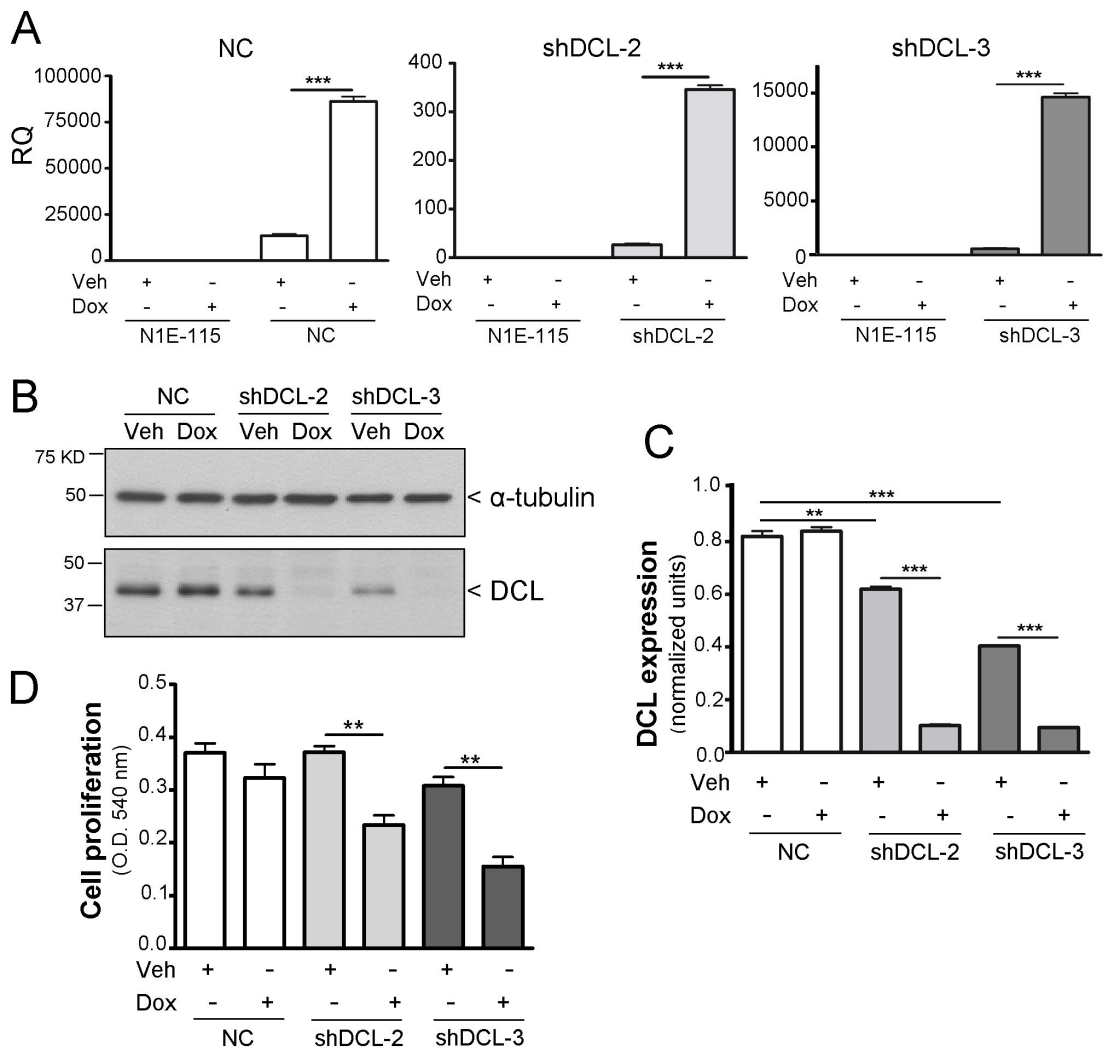
DCL knockdown in Dox-inducible NB cells results in less NB proliferation. (A) shRNA expression in the different NB cell lines treated with doxycycline (Dox) or vehicle (Veh) for 72 hours. (B) Western blotting results of DCL and α-tubulin expression in Dox-inducible NB cells treated for 72 hours with doxycycline (Dox) or vehicle (Veh). (C) Quantification and normalization of DCL expression to α-tubulin. (D) Cell proliferation 72 hours after starting adding Dox or Veh to the growth medium. N1E-115 cells, mouse NB cell line used to develop the Dox-inducible NB cell lines. NC, negative control Dox-inducible NB cells. shDCL-2 and shDCL-3, Dox-inducible NB cell lines that express shRNA against DCL. RQ, relative quantification. Error bars, s.e.m. **, *P*<0.01; ***, *P*<0.001.

In order to further investigate the role of DCL in NB proliferation we used the recently developed Dox-inducible NB stable cell lines that express shRNAs targeting specifically DCL (shDCL-2 and shDCL-3) [9]. For the present study we developed a Dox-inducible NB stable cell line expressing a scrambled shRNA as negative control (NC; Materials and methods). After induction with Dox, all inducible NB stable cell lines expressed significantly more shRNA (*P*<0.001) than cells treated with vehicle (Veh, Figure 1A). No shRNA was detected in the parental NB cell line (N1E-115). The expression of the specific shRNAs resulted in significant DCL knockdown (85.6 ± 2.1% and 76.7 ± 1.5% knockdown in shDCL-2 and shDCL-3, respectively) while no effect on DCL expression was detected in the NC cell line, as compared to the Veh-treated cells, non-induced control (Figure 1B, C). In agreement with low shRNA expression in vehicle-treated cells (Figure 1A), we detected that DCL expression was significantly downregulated in Veh-treated shDCL-2(*P*<0.01) and shDCL-3(*P*<0.001) compared to NC cells, although to a much lesser extent (Figure 1B, C). This reduction in DCL expression levels, in the absence of Dox induction, did not result in any significant effect in cell proliferation. However, treatment with Dox (1 µg/ml, 72 hours) resulted in significant reductions in cell proliferation in shDCL-2 and shDCL-3 (*P*<0.01, vs. Veh-treated cells) but not in NC cells (Figure 1D). In agreement with our previous observations in NB cell lines transiently transfected with synthetic siRNAs [9], these results indicate that high levels of DCL knockdown are required to reduce cell proliferation. Consistently, a panel of four proliferation-related genes [32,33,34,35] nucleolin (Ncl), neudesin (Nenf), pituitary tumor-transforming 1 (Pttg1) and S100 calcium binding protein A6 (S1006) was found downregulated only after Dox-induced DCL knockdown in shDCL-2 and shDCL-3 cell lines (Figure S1). These genes were also found differently expressed in NB cells with DCLK-derived MAPs knockdown [9].

### DCL silencing results in a significant reduction in NB tumor growth

**Figure 2:**
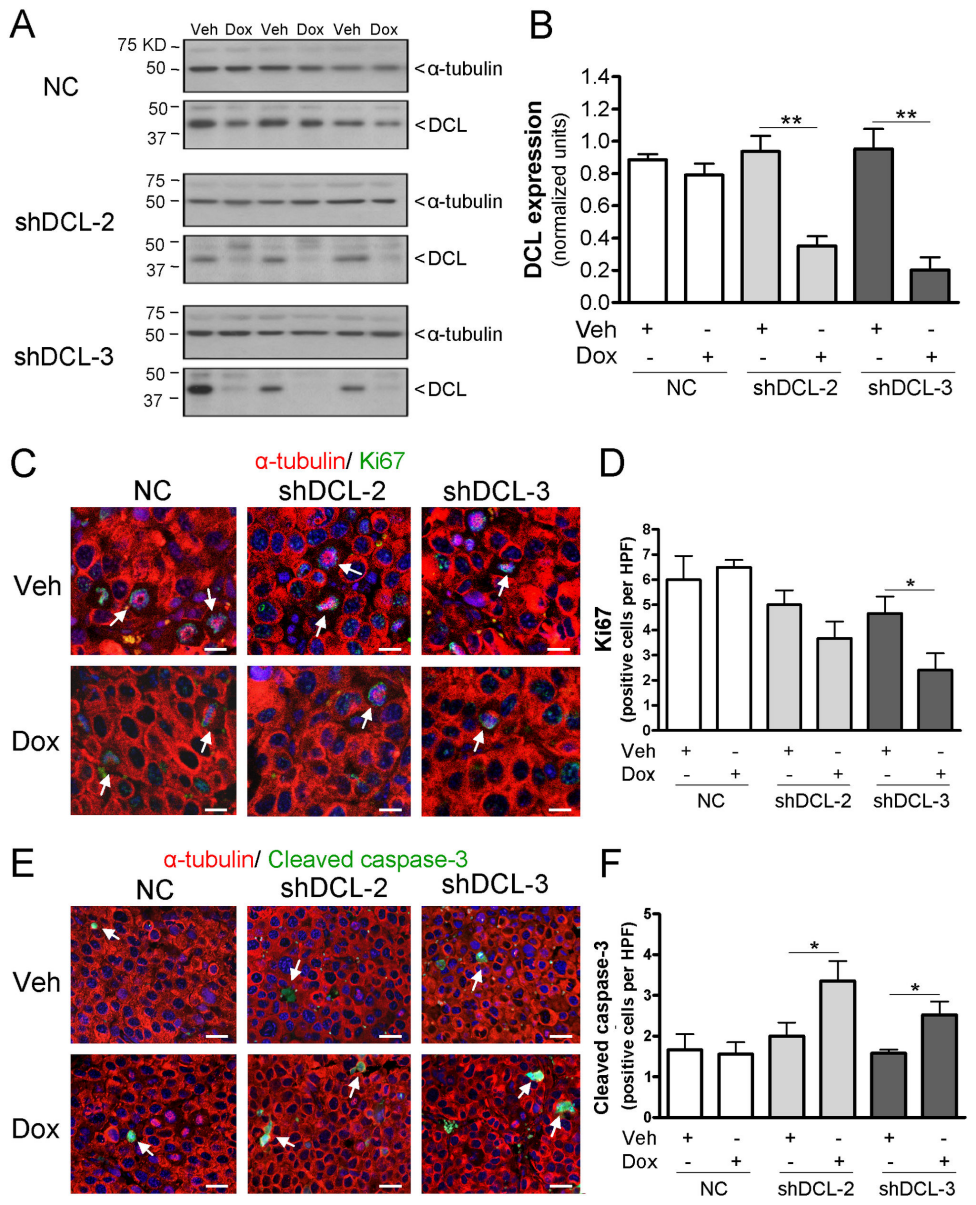
DCL knockdown in Dox-inducible NB tumors correlates with less proliferation and enhanced apoptosis. (A) DCL and α-tubulin expression in Dox-inducible tumors from nude mice treated with doxycycline- (Dox) or vehicle (Veh)-diet. (B) DCL expression normalized to α-tubulin. (C) Immunostaining of Ki67 (green) positive cells in the Dox-inducible NB tumors. (D) Average of positive Ki67 cells per high-power field (HPF). Immunostaining (E) and quantification (F) of cleaved caspase-3 positive cells (green) per HPF. Images (C and E) are representative of the average expression of each group. For each section, 5-10 different fields were analyzed. NC, negative control Dox-inducible NB tumors. shDCL-2 and shDCL-3, Dox-inducible NB tumors that express shRNA targeting DCL. Blue, Hoechst staining. Red, α-tubulin staining. Arrows, examples of Ki67 positive cells (C) or cells with cleaved caspase-3 (E). Error bars, s.e.m. *, *P*<0.05. Scale bars, 25 µm (C) or 50 µm (D).

To study the possible effect of DCL downregulation in NB cells on tumor growth *in vivo*, we developed a mouse NB xenograft model, using the Dox-inducible cell lines shDCL-2 and shDCL-3 (Materials and methods). We observed a significant increase in shRNA expression in tumors after Dox induction (200 mg per kg of diet, 14 days), while shRNAs were below detection levels in tumors from Veh-treated animals (Figure S2). Subsequently, we detected a significant reduction in DCL expression in tumors after Dox-induced shRNA expression (61.3 ± 9.9% and 77.6 ± 7.1% DCL knockdown in Dox-induced shDCL-2 and shDCL-3 respectively, relative to Veh-treated groups). No significant differences were observed in DCL expression between tumors present in animals xenografted with NC, shDCL-2, and shDCL-3 and fed with Veh-diet (Figure 2A, B).

Cell proliferation within the tumor tissue was assessed by immunofluorescence to detect the proliferation marker Ki67 (Figure 2C, D). A significantly reduction (*P*<0.05) in the numbers of Ki67 positive cells was detected only in Dox-treated shDCL-3 tumors. A similar trend was observed in Dox-treated shDCL-2 tumors, although the differences were not statistically significant (Figure 2C, D). Interestingly, a significant increased (*P*<0.05) in cleaved caspase-3 expression was observed in Dox-treated shDCL-2 and shDCL-3 tumors (Figure 2E, F). These results are in agreement with our previous observations that silencing of DCL in NB cells *in vitro* results in induction of apoptotic cell death [9].

**Figure 3:**
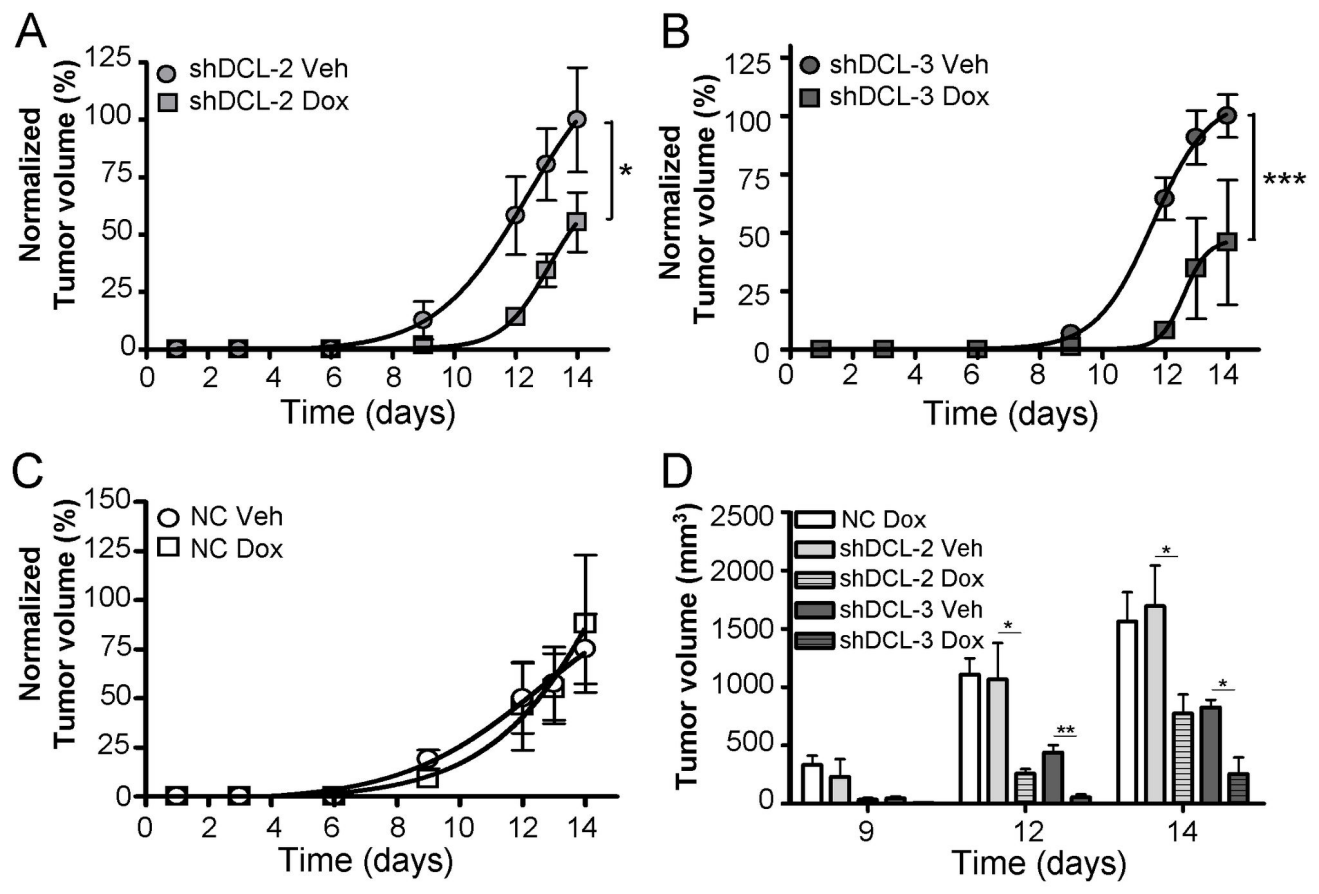
DCL knockdown results in a delayed NB tumor growth. The normalized tumor growth is shown for shDCL-2 (A), shDCL-3 (B) and NC (C) tumors developed from the correspondent Dox-inducible NB cells lines injected subcutaneously in BALB/c athymic nude mice. Tumor growth (A-C) was normalized to the maximum tumor size per group. (D) Average of tumor volume 9, 12 and 14 days after injecting the Dox-inducible NB cells. NC, negative control Dox-inducible NB tumors. shDCL-2 and shDCL-3, Dox-inducible NB tumors that express shRNA against DCL. Error bars, s.e.m. *, *P*<0.05; **, *P*<0.01; ***, *P*<0.001.

**Figure 4:**
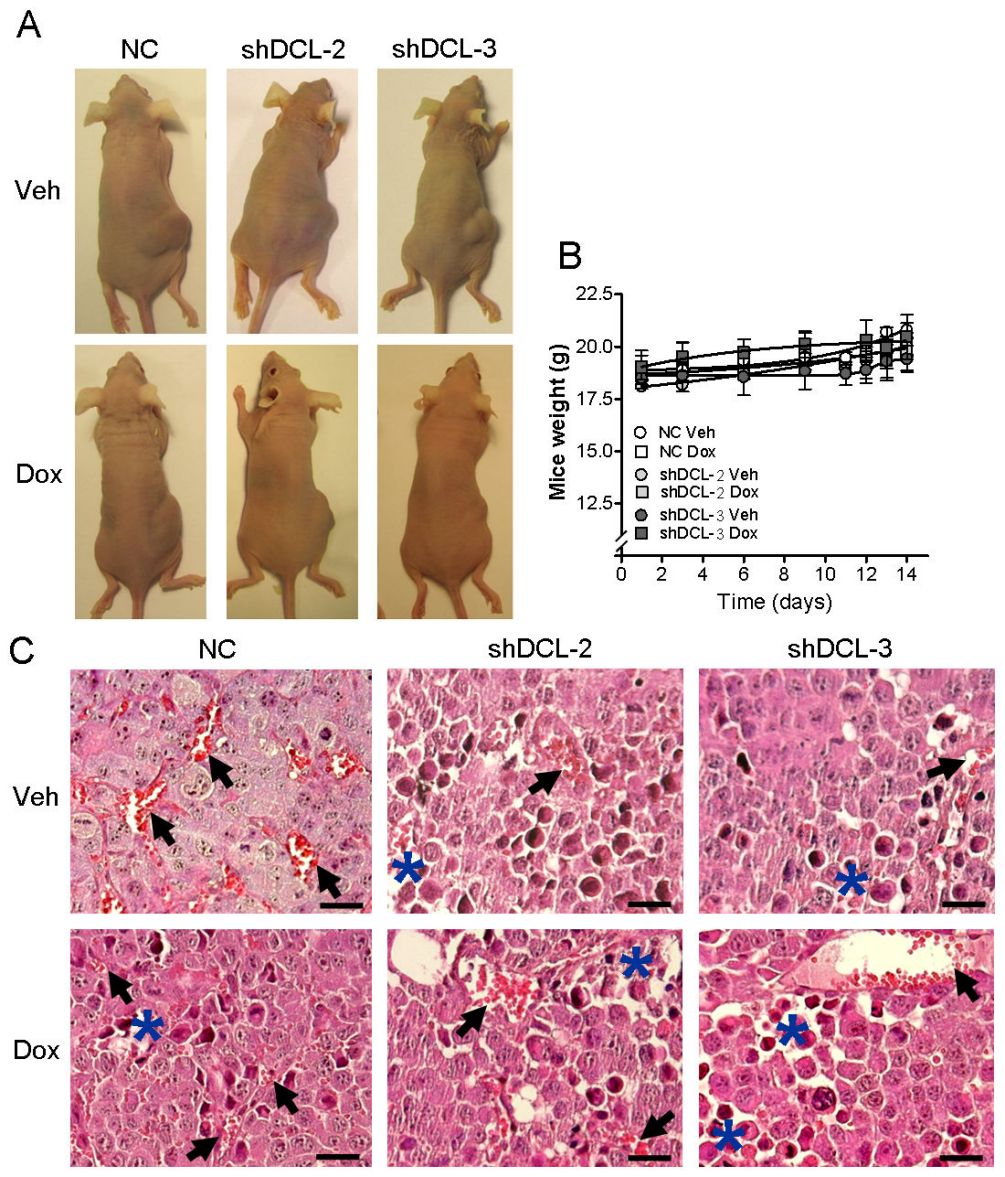
DCL silencing leads to smaller tumor formation with necrotic areas. (A) Representative mice of the different groups 14 days after inoculation of the Dox-inducible NB cells. (B) Mice weight over time. (C) Tumor histology showing high vascularization (arrows) and necrotic areas (* in blue). The quantification of the vascular structures and necrotic areas is shown in Figure S3. Error bars, s.e.m. *, *P*<0.05. Scale bars, 50 µm.

Furthermore, treatment of xenografted animals with Dox (200 mg per kg of diet, 14 days) resulted in a significant reduction in tumor volume after Dox-induced shDCL-2 (F_1,7_ = 5.953; *P*<0.05) and shDCL-3 (F_6,30_ = 27.95; *P*<0.001) expression compared to veh-treatment (Figure 3A, B). Animals xenografted with NC cells did not present significant differences (F_1,8_ = 0.002; *P*=0.969) in tumor volume after Dox or Veh treatment (Figure 3C). We identified a significant reduction in tumor volume from day 12 onwards in animals xenografted with Dox-induced shDCL-2(*P*<0.05) or shDCL-3(*P*<0.01) expressing cells (Figure 3D). Moreover, the first detectable tumors appear at day 9 in Veh-treated groups and at day 12 in Dox-treated groups (Figure 3A, B), suggesting a significant delay in growth in the tumors with reduced amounts of DCL protein. We did not observe any significant difference in body weight arising from Dox or Veh treatment in any experimental group (Figure 4A, B). All tumors presented an undifferentiated histology, necrotic areas and were highly vascularized (Figure 4C and Figure S3).

### DCL colocalizes with mitochondria and DCL knockdown results in decreased expression of mitochondrial-related genes in NB cells

To identify the mechanisms responsible for the effects of DCL downregulation on tumor growth observed *in vivo*, we performed a series of *in vitro* studies using the Dox-inducible shDCL-2, shDCL-3 and NC cell lines. Mitochondria have been shown to be crucial for tumor development [36] and our previous studies identified a highly significant correlation between DCL expression and mitochondrial-related genes [9]. Therefore, we have tested the hypothesis that DCL is involved in the regulation of mitochondrial functioning in NB cell. For this, we investigated possible DCL colocalization with mitochondria and the effects of DCL downregulation on the expression of a set of mitochondrial-related genes.

**Figure 5:**
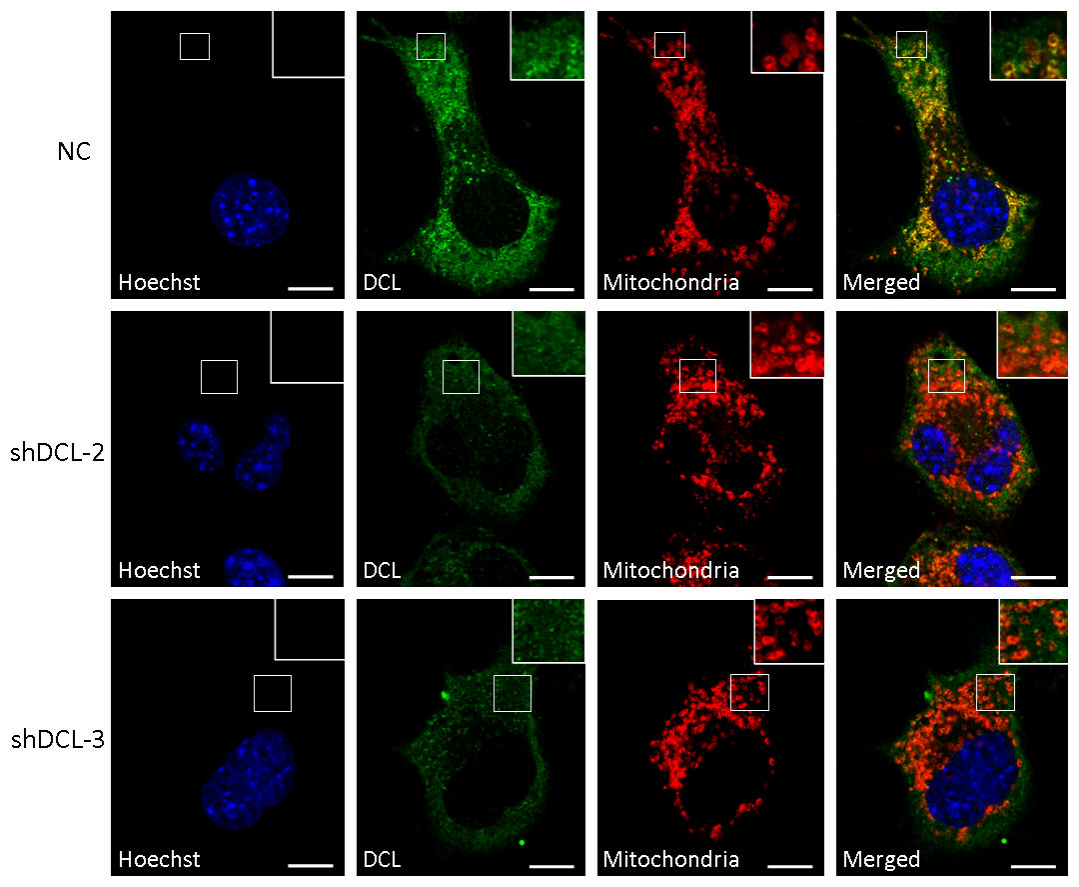
DCL knockdown results in less mitochondrial colocalization in NB cells. DCL (green) and mitochondria (red) staining in Dox-inducible NB cells treated with doxycycline revealed less colocalization (yellow) in NB cells with DCL knockdown (shDCL-2 and shDCL-3). Colocalization scores and images of Dox-inducible NB cells treated with vehicle are shown in Figure S4. Mitochondria were stained with 100 nM MitoTraker Orange CMTM Rosamine. Blue, Hoechst staining. NC, negative control Dox-inducible NB cell lines that express scramble shRNA. shDCL-2 and shDCL-3, Dox-inducible NB cell lines that express shRNA against DCL. Scale bars, 10 µm.

**Figure 6:**
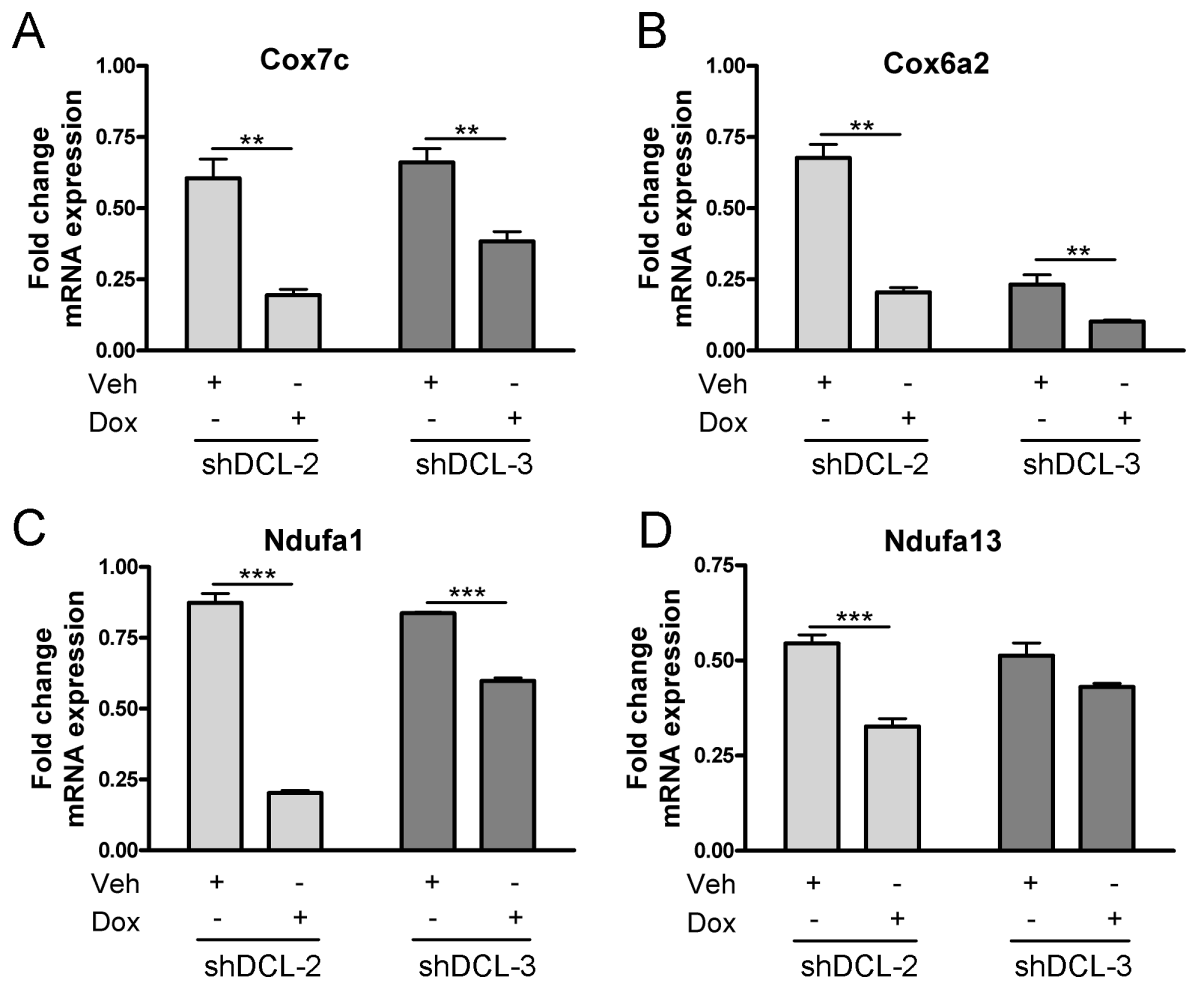
DCL knockdown leads to a lower expression of mitochondrial-related genes. Fold change of Cox7c (A), Cox6a2 (B), Ndufa1 (C) and Ndufa13 (D) mRNA expression in Dox-inducible NB cells (shDCL-2 or shDCL-3). Cells were treated with doxycycline (Dox) or vehicle (Veh) for 72 hours. Fold change was calculated by normalization to the negative control (NC) Dox-inducible NB cell line treated with Dox or Veh respectively. Error bars, s.e.m. **, *P*<0.01; ***, *P*<0.001.

Previous studies predicted a mitochondrial location for DCL [9]. In the present study we found a substantial colocalization between DCL and mitochondria in NB cells (Figure 5 and Figure S4). Using a mouse adult brain library in a yeast two-hybrid screen, we found that the C-terminal domain of DCL interacts with the mitochondria outer membrane protein 25 (OMP25)/ synaptojanin-2-binding protein (SYNJ2BP) [37] (Table 1), consistent with the colocalization results shown in Figure 5 and Figure S4. Interestingly, induction of DCL downregulation by Dox treatment in shDCL-2 and shDCL-3 cells resulted in a significant reduction (*P*<0.05) in DCL/mitochondria colocalization score (Figure S4). No significant differences in colocalization score were found between Veh-treated shDCL-2, shDCL-3 and NC cells and Dox-treated NC cells (Figure S4). Further, we found significant downregulation of the mitochondrial-related genes cytochrome c oxidase, subunit VIIc (Cox7c); cytochrome c oxidase, subunit VIa (Cox6a2) and NADH dehydrogenase (ubiquinone) 1 alpha subcomplex, 1 (Ndufa1) in both shDCL-2 and shDCL-3 NB cells treated with Dox (*P*<0.01 or *P*<0.001, Figure 6A-C). In addition, NADH dehydrogenase (ubiquinone) 1 alpha subcomplex, 13 (Ndufa13 or GRIM-19) was significantly downregulated in Dox-treated shDCL-2 cells, while a trend towards less Ndufa13 expression was observed in Dox-treated shDCL-3 cells (Figure 6D). These data suggests that DCL is involved in mitochondrial activity regulation.

**Table 1:** Protein preys interacting with the C-terminal domain of DCL in a yeast two-hybrid screen, using an adult mouse brain library.

**Protein**	**Global PBS**	**Clones**	**SID**	**Full length**
Synj2bp	A	5	1-145	145
Ap1m1	D	3	118-423	423
Gnl3l	D	1	8-268	577
Zfp106	E	1	1420-1658	1888
Zfp521	E	4	51-225	1311

Note: The confidence in the interactions was categorized according to global PBS scores. PBS A-D indicates very high, high, good and moderate confidence in the interaction, respectively. E denotes highly connected protein domains (high likelihood of artifacts). High confidence binding was observed to the outer membrane mitochondria protein Synj2bp/OMP25 (PBS = A). Five independent prey fragments (clones) identified the interaction, all encoding the full-length Synj2bp protein of 145 amino acids. Moderate or highly connected interactions were indicated to prey fragments matching four additional reference proteins (PBS = D/E). Abbreviations: PBS, proteome-wide interaction map(PIM) biologic score; SID, selected interaction domain (amino acid sequence) shared by all prey fragments matching the same reference protein; Full length, number of amino acids in reference protein.

### DCL regulates mitochondrial activity in NB cells

**Figure 7:**
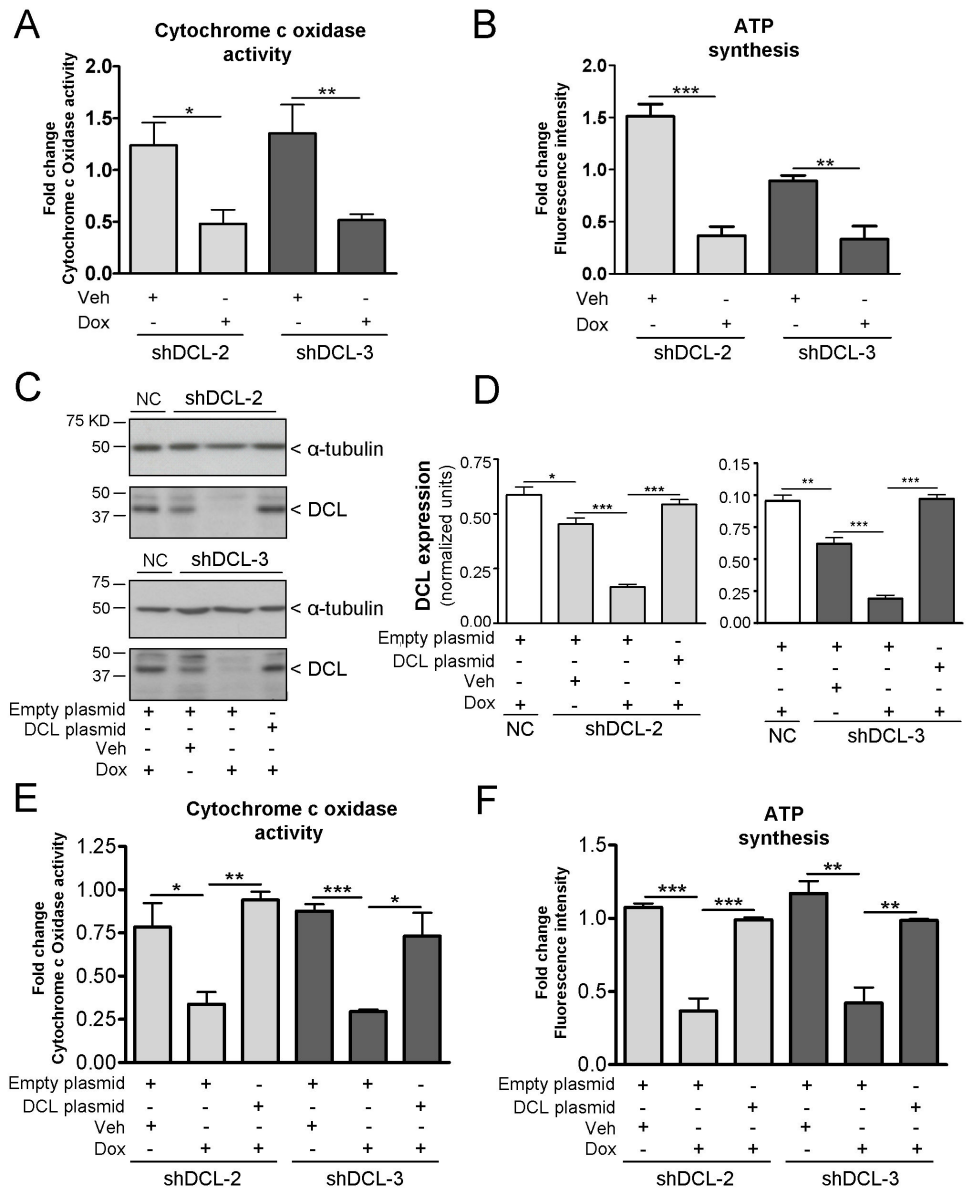
DCL plays a role in mitochondrial activity. Fold change in cytochrome c oxidase activity (A) and ATP synthesis (B) in shDCL-2 and shDCL-3 Dox-inducible NB cells treated for 72 hours with doxycycline (Dox) or vehicle (Veh). (C) DCL and α-tubulin expression in Dox-inducible NB cells treated with either Dox or Veh for 72 hours and subsequently transfected with DCL or empty plasmids. Cells were lysed for western blotting analysis 48 hours after transfection. (D) DCL expression normalized to α-tubulin. DCL expression is recovered in shDCL-2 and shDCL-3 cells after transfection with DCL plasmid (C-D). Fold change of cytochrome c oxidase activity (E) and ATP synthesis (F) in shDCL-2 and shDCL-3 cells treated with either Dox or Veh (72 hours) and then transfected with DCL or empty plasmid (48 hours). Fold changes were determined by normalizing to the negative control (NC) Dox-inducible NB cell line treated with Dox or Veh respectively. Cytochrome c oxidase activity was investigated in isolated mitochondria. Error bars, s.e.m. *, *P*<0.05; **, *P*<0.01; ***, *P*<0.001.

To test a DCL role in mitochondrial activity, we investigated the activity of cytochrome c oxidase. Mitochondria isolated from NB cells with Dox-induced DCL knockdown showed significantly less cytochrome c oxidase activity (*P*<0.05 in shDCL-2 and *P*<0.01 in shDCL-3, Figure 7A). Moreover, we found that ATP synthesis was significantly inhibited (*P*<0.01) after DCL knockdown in shDCL-2 and shDCL-3 cells (Figure 7B). Notably, transfection of shDCL-2 and shDCL-3 cells with a shRNA resistant DCL plasmid (Materials and methods) recovered DCL expression, cytochrome c oxidase activity and ATP synthesis, suggesting that DCL is crucially involved in controlling mitochondrial activity and energy production in NB cells (Figure 7C-F).

### Glucose restriction results in reduction of NB cell proliferation after DCL downregulation

**Figure 8:**
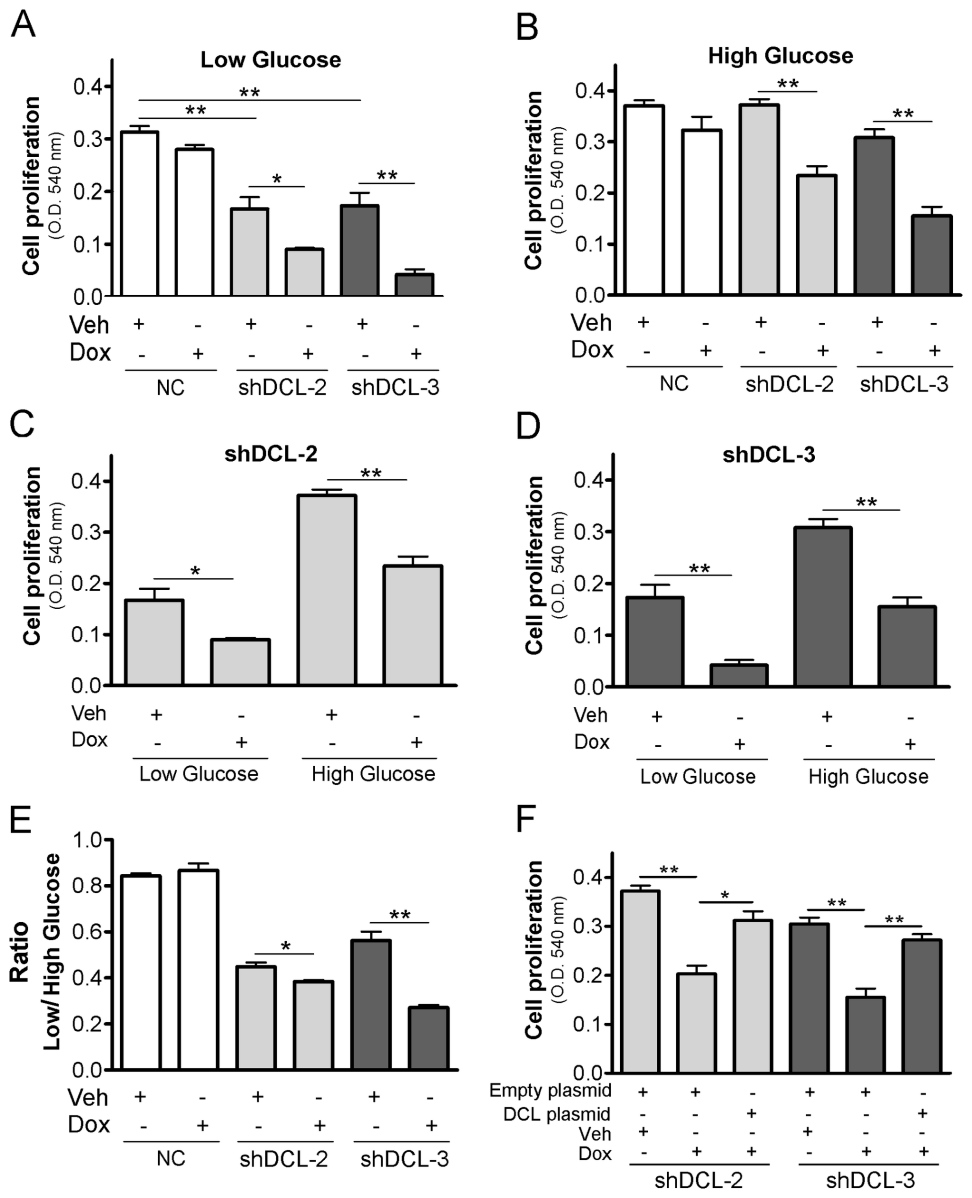
NB cells with DCL knockdown proliferate less in an energetic challenged environment. (A-D) Cell proliferation in Dox-inducible NB cell lines in the presence of low glucose (A) or high glucose (B) medium and treated with doxycycline (Dox) or vehicle (Veh) for 72 hours. Cell proliferation in shDCL-2 (C) and shDCL-3 (D) cells 72 hours after starting the treatment with either Dox or Veh and in the presence of low or high glucose medium. (E) Ratio in cell proliferation between Dox-inducible NB cells that grew in low and high glucose medium. (F) Cell proliferation in shDCL-2 and shDCL-3 cells grown in high glucose medium, treated with Dox or Veh for 72 hours and subsequently transfected with empty plasmid or DCL plasmid to recover DCL expression. NC, negative control Dox-inducible NB cell lines. shDCL-2 and shDCL-3, Dox-inducible NB cell lines that express shRNA against DCL. Error bars, s.e.m. *, *P*<0.05; **, *P*<0.01.

In the presence of oxygen, most cells produce energy from glucose by OXPHOS in mitochondria. This OXPHOS-regulated energy is crucial for cell proliferation [20,36]. Nevertheless, many tumor cells, including NB, also process glucose by aerobic glycolysis [38,39]. In order to understand whether the deficits in mitochondrial activity were associated with DCL knockdown in NB cells, we exposed NB cells to glucose restriction and analyzed the effects of DCL downregulation on cell proliferation. Proliferation of shDCL-2 and shDCL-3 cells was reduced under low glucose conditions (Figure 8A) with or without Dox treatment. This effect was not observed in NC cells. Importantly, proliferation was significantly inhibited by DCL downregulation in both low and high glucose environments (*P*<0.05, Figure 8A-D). We did not observe significant differences in the low/high glucose proliferation ratio in NC cells. However, Dox-treated shDCL-2 and shDCL-3 cells presented a significantly reduced low/high glucose proliferation ratio (*P*<0.05 in shDCL-2 and *P*<0.01 in shDCL-3, Figure 8E). These results suggest that after DCL downregulation NB cells proliferate less in an energy supply-restricted environment. To support this conclusion, rescue of DCL expression in the Dox-inducible cell lines results in a concomitant recovery of cell proliferation. Interestingly, recovery of DCL expression also resulted in a significant decrease in caspase-3 activity (Figure S5).

### S/P-rich and the second DCX-domains of DCL are required for mitochondrial activity regulation

**Figure 9:**
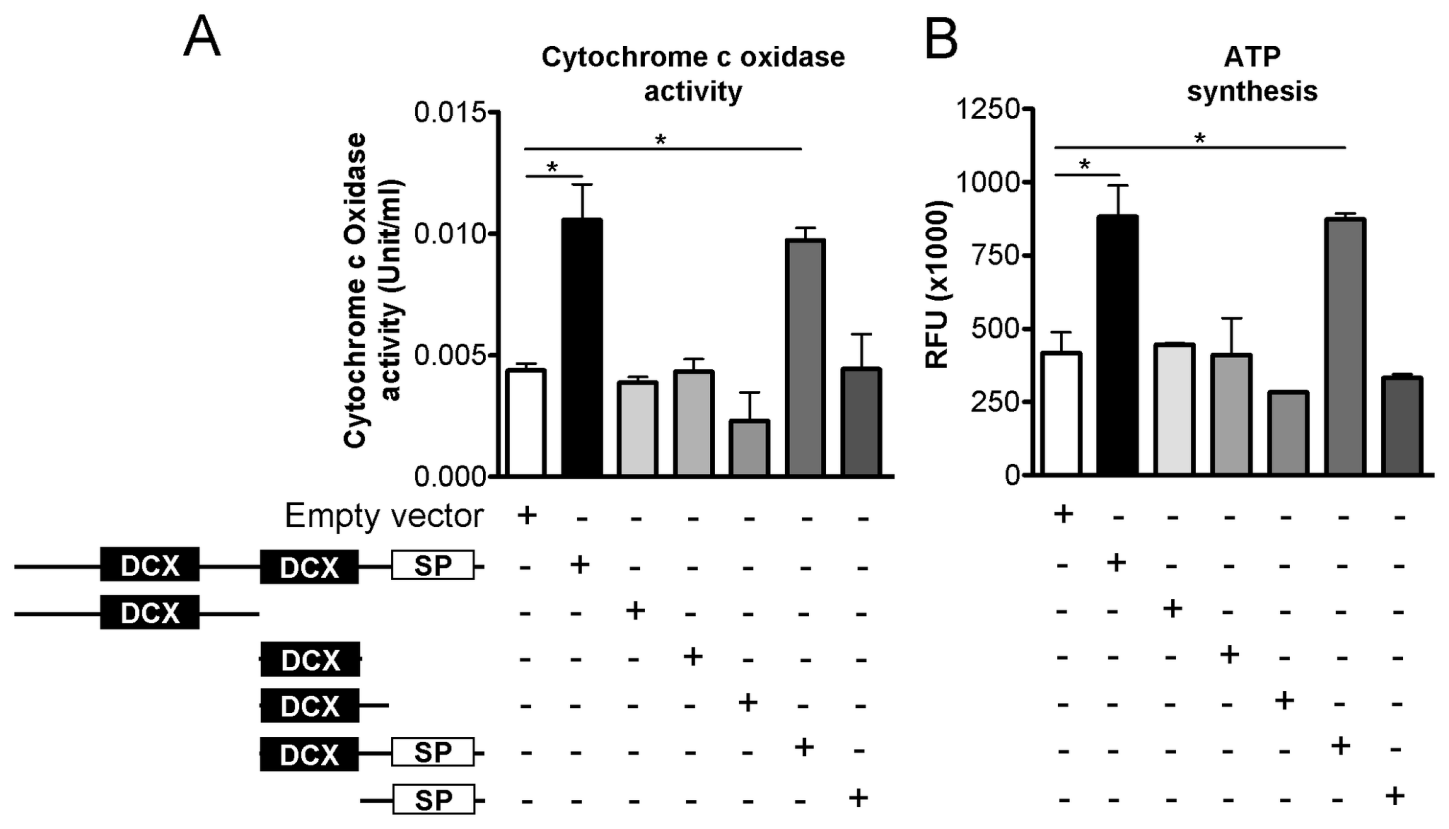
Serine/Proline (S/P)-rich and the second doublecortin (DCX)-domains of DCL are required for mitochondrial activity regulation. Cytochrome c oxidase activity (A) and ATP synthesis (B) in COS-1 cells transfected with empty vector, with DCL full-length or with one of the different DCL fragments subcloned into pDsRed2-N1 vector is shown. The structure of DCL full-length and the different DCL mutants is represented, indicating the DCX-domains and S/P-rich domain (SP). Error bars, s.e.m. *, *P*<0.05.

Previous studies have demonstrated that proteins derived from the *DCLK1* gene present microtubule binding-dependent and independent functions [13,14]. Furthermore, results from out yeast two-hybrid screen suggested that C-terminal domain of DCL interacts with the mitochondria outer membrane proteins (Table 1). In a primary effort to characterize the DCL protein domains involved in regulation of mitochondrial activity, we developed seven DCL mutants containing different structurally relevant combinations of the two DCX-domains and the C-terminal S/P-rich domain (Material and methods). Subsequently, we induced their expression in COS-1 cells (Figure S6) and studied the effect on microtubule bundling, intracellular localization, cytochrome c oxidase activity and ATP synthesis (Figure 9, Figure S7 and Table S2).

Interestingly, significantly increased (*P*<0.05) cytochrome c oxidase activity and ATP synthesis was detected in COS-1 cells transfected with the full-length DCL (Figure 9). These increases in cytochrome c activity and ATP synthesis were only replicated by a mutant containing both the second DCX-domain and the S/P-rich domain (Figure 9). No significant differences in cytochrome c oxidase activity or ATP synthesis were detected between cells transfected with the empty pDsRed2-N1 vector and any other mutant (Figure 9A, B). Full-length DCL overexpression in COS-1 cells induces strong DCL colocalization to microtubules and microtubule bundling; most of DCL’s known biological activities have been linked to this property [10,11,13]. Therefore, we investigated the cellular location and the microtubule bundling activity of the six DCL mutants that we were able to express in COS-1 cells. We found that the second DCX-domain and the linker between this domain and the S/P-rich domain are the minimal domains required for microtubule bundling (Figure S7).

Our preliminary characterization of the protein domains required for regulation of mitochondrial activity indicated that both the second DCX-domain and the S/P-rich domain are necessary for this new biological activity of DCL. As the presence of the second DCX-domain, the S/P-rich domain and the short linker between these two domains are sufficient to induce microtubule bundling in COS-1 cells, we conclude that the regulation of mitochondrial activity may be linked to DCL’s microtubule-binding activity.

## Discussion

In the present study we show that downregulation of the MAP DCL results in inhibition of neuroblastoma (NB) cell proliferation *in vitro* and in delayed NB tumor development *in vivo*. Further, we demonstrate that DCL colocalizes with mitochondria, interacts with the mitochondrial outer membrane protein SYNJ2BP/OMP25 and is involved in the regulation of mitochondrial activity and ATP synthesis. Moreover, we map this new biological activity to the C-terminal domains of the DCL protein, specifically to DCL’s second DCX-domain and its S/P rich domain. Therefore, our data reveal a novel function for DCL, i.e. regulation of mitochondrial activity and ATP synthesis, which may contribute to the regulation of NB tumor growth.

We observed a significant delay in NB xenograft tumor growth in mice with induced DCL knockdown. Recently, we and others have shown that DCL is a crucial MAP for mitotic spindle formation and stabilization and as such is vital for neuroblast proliferation and migration [10,11]. Moreover, DCL knockdown results in cell-cycle arrest and apoptosis in neuroblasts and NB cells [9,10]. The results of the present study indicate that DCL plays a role in NB tumorigenesis and that DCL loss-of-function results in inhibition of NB proliferation *in vitro* and *in vivo*.

Tumors with DCL knockdown were detected at a later time point and remained significantly smaller than tumors expressing higher DCL amounts. However, we did not observe a complete arrest of tumor growth. It has been suggested that long-term suppression of a certain gene/protein can result in alternative and/or compensatory mechanisms that allow the cells to proliferate [40,41]. This might be the reason why, even when significantly delayed, we detected tumor development in the presence of lower DCL expression levels. DCLK-long is another MAP encoded by *DCLK1* gene that plays a role in microtubule stabilization and neuroblast proliferation [10,11]. Both DCL and DCLK-long are highly expressed in NB and in glioblastoma [9], indicating that these MAPs play a role in the development in these tumor types. Recent studies have shown that DCLK1 is highly expressed in gastrointestinal stem cells and it marks tumor stem cells that produce tumor progeny in the polyps of Apc (Min/+) mice [42,43,44], suggesting an important function for DCLK1 in colorectal cancer as well. Overexpression of DCLK1 has also been detected in breast, pancreas and prostate tumors [42,43,44]. Therefore, it would be of great interest to further investigate whether or not DCLK-long compensates for DCL knockdown and which potential compensatory mechanisms were involved. This may allow exploring new combinations of therapeutic approaches. Previous studies have shown that combining silencing of DCLK-derived MAPs with microtubule destabilizing agents, such as vinca alkaloids, results in synergistic apoptotic effect [45]. This may be an attractive approach to explore *in vivo* as well. Moreover, in the present study, tumors that expressed high levels of DCL revealed fewer apoptotic cells. This finding is in agreement with previous reports in NB cell lines showing that DCL knockdown leads to apoptotic cell death [9,45].

Because mitochondrial activity and energy production is of high importance for cell proliferation and tumor growth [17,46,47,48,49,50], we investigated the link between mitochondrial regulation and DCL expression. We found a significant downregulation of several mitochondrial-related genes in NB cells with DCL knockdown which might result from disruption of the mitotic spindles [10,11] and from the disturbance of protein translocation to the nucleus through the microtubules. Previous studies have shown that DCL regulates the translocation of the glucocorticoid receptor (GR), a receptor crucially involved in energy metabolism, to the nucleus in neuronal progenitor cells and in neuroblastoma cells [13]. Once in the nucleus, GR regulates the expression of several genes, including mitochondrial-related genes [51]. In the present study, besides the changes in gene expression, we have also detected alterations in mitochondrial activity and reduction of ATP synthesis after DCL knockdown, suggesting that DCL plays a role in mitochondrial activity. These effects were restored when we rescued DCL expression in NB cells with DCL knockdown. Moreover, we observed a clear DCL colocalization with mitochondria and a direct interaction between the C-terminal domain of DCL and the mitochondrial outer membrane protein OMP25/ SYNJ2BP [37]. In line with this are our previous data reporting DCL expression in human NB tumors, which correlates significantly with the expression of mitochondrial-related genes, including genes that are involved in the oxidative phosphorylation (OXPHOS) process [9]. Also, based on its primary amino acid sequence, DCL is predicted to localize in mitochondria [9]. However, further research is necessary to investigate whether DCL is indeed localized in mitochondria, if it interacts with the integral mitochondrial outer membrane protein OMP25/ SYNJ2BP and if that would be related with the regulation of mitochondrial activity.

DCL may regulate NB tumor growth by linking stabilization of mitotic spindles, mitosis of NB cells and mitochondrial activity. It has been demonstrated that mitotic stimuli lead to the activation of mitochondrial bioenergetic processes by transcription activation of mitochondrial genes [15,16]. Proliferation in cancer cells has been associated with increased rates of OXPHOS and glycolysis [17]. By exposing NB cells to growth in low glucose conditions, we were able to observe that NB cells with DCL knockdown were less able to cope with this energetic challenge. This is in agreement with our results that show less cytochrome c oxidase activity and lower ATP levels synthesized by NB cells with DCL knockdown. Therefore, the present data suggest that mitochondrial energy processing pathways regulated by DCL are rate-limiting during NB proliferation. Several tumor cells, including NB, switch to a preference for glucose metabolic processing by aerobic glycolysis [17,18], a process rarely observed in non-transformed cells, known as the Warburg effect [52,53]. Therefore, targeting the glycolytic process has been proposed as therapeutic approach for cancer [49]. However, it was observed that inhibition of glycolysis alone is not sufficient to fully inhibit cell proliferation and tumor development because cells are still able to produce ATP via OXPHOS [49]. Therefore, combining glycolytic inhibitors with DCL loss-of-function could be an effective approach for future NB treatment. In the same line of reasoning, recent studies have demonstrated that fasting cycles retard tumor growth and sensitize many cancer cells to chemotherapy, including NB, by increasing oxidative stress, caspase-3 cleavage, DNA damage and apoptosis [54].

Structurally, we demonstrate that the C-terminal portions of DCL containing the second DCX and the S/P-rich domains are required for cytochrome c oxidase activity and ATP synthesis. Nevertheless, we were unable to separate the newly observed regulation of mitochondrial activity from the previously observed regulation of microtubule processing by DCL, suggesting that these two different biological activities may be linked. Indeed, there are indications that phosphorylation of the S/P-rich domain of DCX family members is involved in regulating cytoskeleton dynamics [14] and an intact microtubule network together with MAPs is required for mitochondrial transport throughout the cell [55,56] and to regulate mitochondrial function [57,58,59,60]. It has been shown that the interaction between MAP2 with the outer mitochondrial membrane protein porin, induces alterations in the physicochemical properties of porin environment [57,60]. The dual mitochondria and microtubule related functions have been shown also in other MAPs, such as RASSF1 [61,62,63], C19ORF5, also called MAP1S or RABP1 [61,62,63], and SPD-3 [64].

The S/P-rich domain has been proposed as a protein interaction platform [14]. DCL interact with the nuclear receptor glucocorticoid receptor via the C-terminal SP-rich domain [13] and therefore its presence might be required for regulation of mitochondrial-related genes expression [51]. Furthermore, it has been demonstrated that S/P-rich domain would be phosphorylated by CDK5, protein kinase A (PKA), mitogen-activated protein kinase (MAPK), c-Jun N-terminal kinases (JNK) and phosphatases PP1 and PP2 [14,65,66]. Glycogen synthase kinase (GSK-3) and MAPK extracellular signal-regulated protein kinase 1 (ERK1) have been predicted to phosphorylate S/P-rich domain as well [14]. These kinases that regulate the dynamic phosphorylation state of the S/P-rich domain present in several MAPs of the DCX family are known to play a role in cell proliferation [14,67]. Although we cannot confirm that the link between DCL and mitochondrial activity is related to mitochondria transport along microtubules, our study clearly shows that the S/P-rich and the second DCX-domain are required for proper mitochondrial activity in NB cells. These domains are also present in other proteins of the DCX family, such as DCX and DCLK2 [14]. It would be of interest to further investigate if these proteins are also expressed in NB and regulate mitochondrial activity and energy production.

Our results show, for the first time a role for DCL in NB tumor growth *in vivo*. Our study reveals that DCL is involved in the regulation of mitochondrial activity and energy production, which are crucial processes for cell proliferation. Moreover, we identify the DCX and S/P-rich domains as required for regulation of mitochondrial activity. As these domains are also present in other members of the DCX family, our results may reveal a novel regulation mechanism involving energy supply necessary for neuronal migration and proliferation of neuronal progenitor cells. Furthermore, it may provide solid bases for future studies such as potential combined therapeutic approaches for NB.

## Supporting Information

Figure S1 **DCL silencing results in down-regulation of proliferation-related genes.** Fold change in Ncl, Nenf, Pttg1 and S1006 mRNA expression in Dox-inducible NB cells (shDCL-2 and shDCL-3) 72 hours after starting doxycycline (Dox) or vehicle (Veh) treatment. Fold change was calculated by normalizing to the negative control Dox-inducible NB cells treated with Dox or Veh respectively. Error bars, S.E.M. *, *P* < 0.05; **, *P* < 0.01; ***, *P* < 0.001.
(TIF)

Figure S2 **Dox-diet induces shRNA expression in Dox-inducible NB tumors.** shRNA expression in the different Dox-inducible NB tumors 14 days after injecting the NB cells subcutaneously. Mice received doxycycline (Dox)- or vehicle (Veh)-diet. NC, negative control Dox-inducible NB tumors. shDCL-2 and shDCL-3, Dox-inducible NB tumors that express a shRNA against DCL. N1E-115 cells, NB cell line used to develop the Dox-inducible NB cells. RQ, relative quantification. Error bars, S.E.M. ***, *P* < 0.001.
(TIF)

Figure S3 **The tumor histology revealed high vascularization and necrotic areas.** (A) Vascular counts per microscopic field (200x magnification). Pictures are randomly taken from three fields each at a magnification of ×200 (H&E staining) from six independent sections. (B) Estimation of the percentage of necrotic areas in the tumors. The necrotic areas were quantified relative to total pixel density. Error bar, S.E.M. *, *P* < 0.05.
(TIF)

Figure S4 **DCL colocalizes with mitochondria.** (A) DCL (green), mitochondria (red) and nuclei (Hoechst, blue) staining in Dox-inducible NB cells treated with vehicle (Veh). (B) Colocalization scores in Dox-inducible NB cells after 72 hours doxycycline (Dox)- or Veh-treatment. Colocalization score was quantified using ImageJ as describes previously (Fitzsimons et al., 2008). NC, negative control Dox-inducible NB cells. shDCL-2 and shDCL-3, Dox-inducible NB cell lines that express a shRNA against DCL. Scale bars, 10 µm. Error bars, S.E.M. *, *P* < 0.05.
(TIF)

Figure S5 **Recovery of DCL expression results in a decrease in caspase-3 activity.** Caspase-3 activity in Dox-inducible NB cells (NC, shDCL-2 and shDCL-3) transfected with DCL or empty plasmid is shown. Transfection was performed 72 hours after starting doxycycline (Dox)- or vehicle (Veh)-treatment and caspase-3 activity was investigated 48 hours after the transfection. Error bars, S.E.M. *, *P* < 0.05.
(TIF)

Figure S6 **Expression of DCL and DCL mutants in COS-1 cells transfected with the different DCL sequences subcloned in pDsRed2-N1 vector.** (A) Schematic representation of DCL full-length (1) and the different DCL mutants (2-7), showing the doublecortin domains (DCX-domains) and Serine/Proline (S/P)-rich domain (SP). (B) Western blotting results showing the expression of the fusion proteins DCL full-length-DsRed2 and DCL truncations-DsRed2. The expression of S/P-rich domain alone was not detectable, as previously reported (Vreugdenhil et al. 2007).
(TIF)

Figure S7 **DCL full-length induces microtubule bundling and is located in the nucleus and cytoplasm.** Immunofluorescence and colocalization of DCL full-length or DCL mutants (red) and nucleus (Hoechst staining, blue) in transfected COS-1 cells with different DCL sequences subcloned into pDsRed2-N1 vector. DCL full-length and the different DCL truncations were found present (+) or absent (-) in the nucleus, cytoplasm and/or in microtubule bundling. The second doublecortin (DCX) domain and the linker between this domain and Serine/Proline (S/P)-rich domain (SP) were found to be needed for microtubule bundling. +/-, present in few cells. Scale bars, 40 µm.
(TIF)

Table S1 (PDF)

Table S2 (PDF)
