# Supplementary figures and images for: Silencing of Doublecortin-Like (DCL) Results in Decreased Mitochondrial Activity and Delayed Neuroblastoma Tumor Growth
Source: PLoS One. 2013 Sep 26;8(9):e75752. doi: 10.1371/journal.pone.0075752 (PMC3784435; doi:10.1371/journal.pone.0075752)

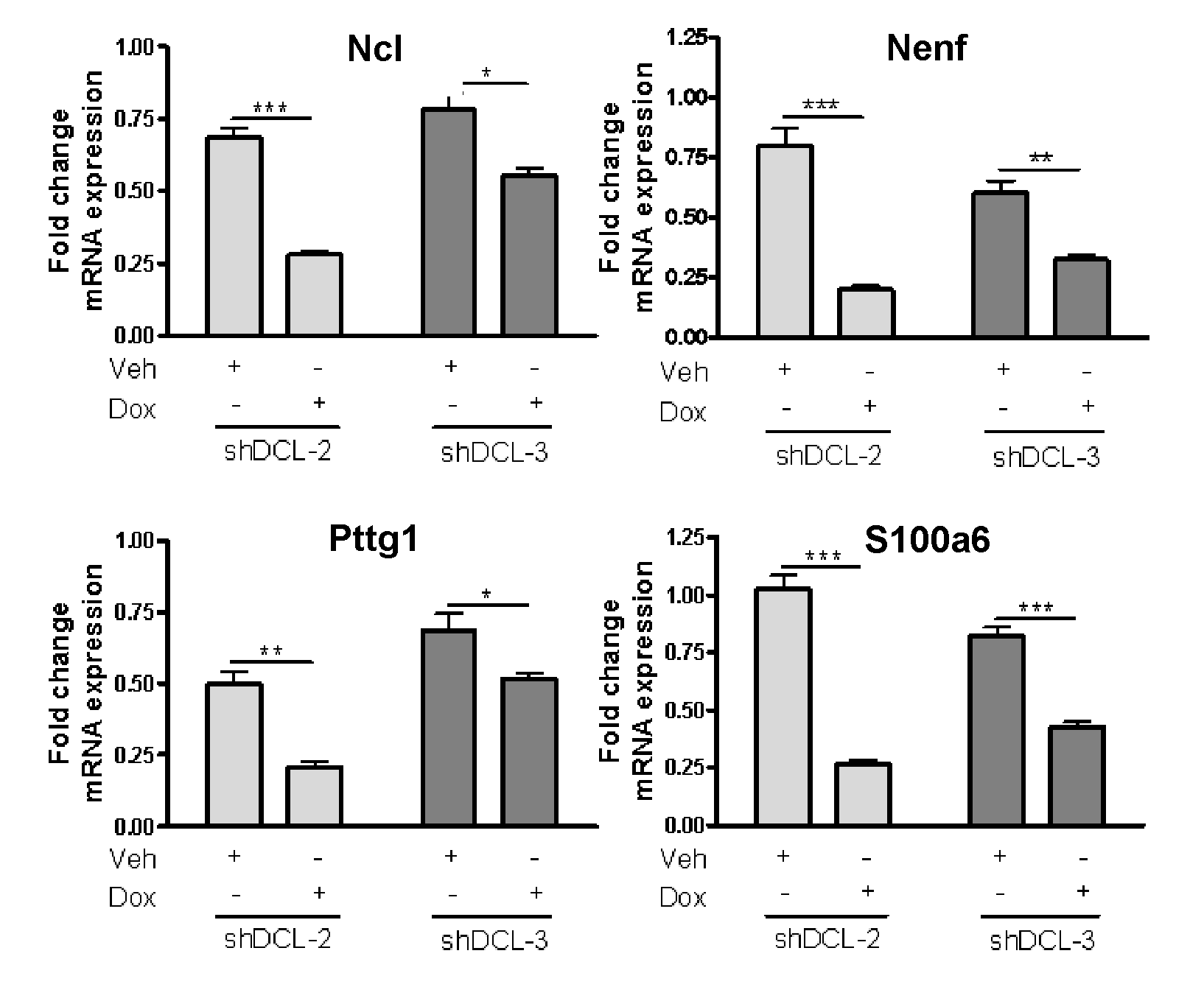

Supplement: Figure S1 — DCL silencing results in down-regulation of proliferation-related genes. Fold change in Ncl, Nenf, Pttg1 and S1006 mRNA expression in Dox-inducible NB cells (shDCL-2 and shDCL-3) 72 hours after starting doxycycline (Dox) or vehicle (Veh) treatment. Fold change was calculated by normalizing to the negative control Dox-inducible NB cells treated with Dox or Veh respectively. Error bars, S.E.M. *, P < 0.05; **, P < 0.01; ***, P < 0.001. (TIF) [file pone.0075752.s001.tif]

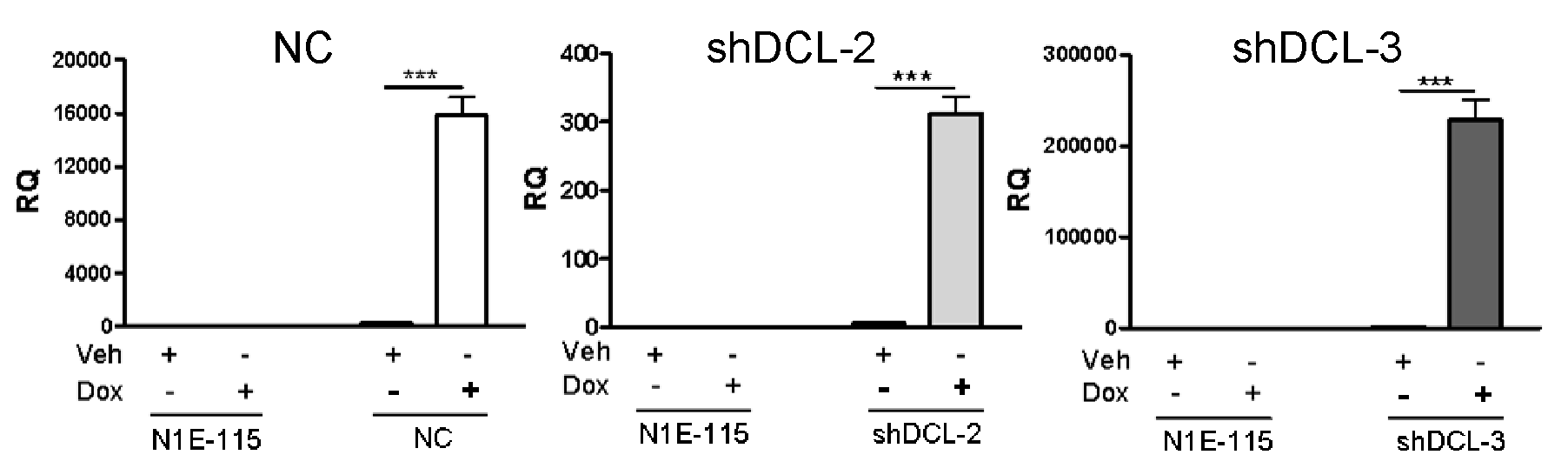

Supplement: Figure S2 — Dox-diet induces shRNA expression in Dox-inducible NB tumors. shRNA expression in the different Dox-inducible NB tumors 14 days after injecting the NB cells subcutaneously. Mice received doxycycline (Dox)- or vehicle (Veh)-diet. NC, negative control Dox-inducible NB tumors. shDCL-2 and shDCL-3, Dox-inducible NB tumors that express a shRNA against DCL. N1E-115 cells, NB cell line used to develop the Dox-inducible NB cells. RQ, relative quantification. Error bars, S.E.M. ***, P < 0.001. (TIF) [file pone.0075752.s002.tif]

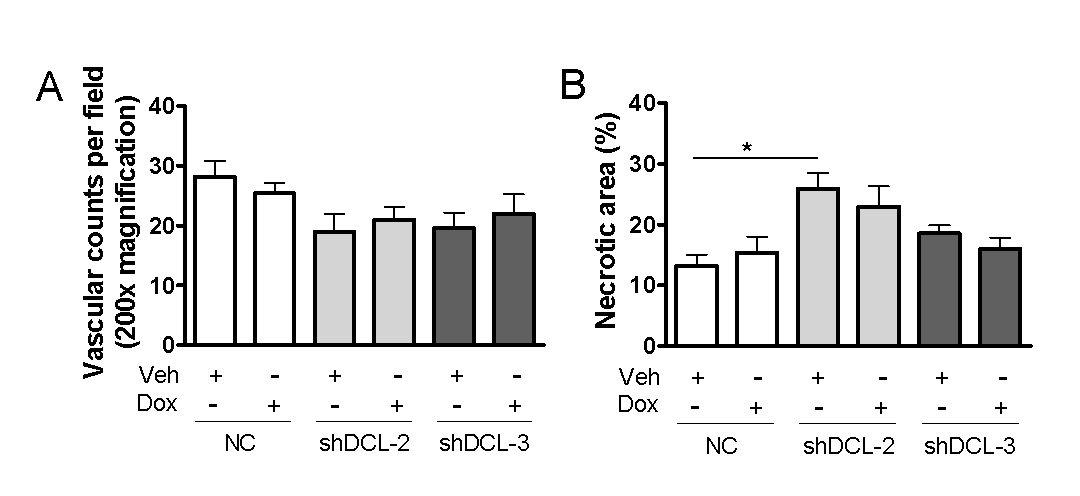

Supplement: Figure S3 — The tumor histology revealed high vascularization and necrotic areas. (A) Vascular counts per microscopic field (200x magnification). Pictures are randomly taken from three fields each at a magnification of ×200 (H&E staining) from six independent sections. (B) Estimation of the percentage of necrotic areas in the tumors. The necrotic areas were quantified relative to total pixel density. Error bar, S.E.M. *, P < 0.05. (TIF) [file pone.0075752.s003.tif]

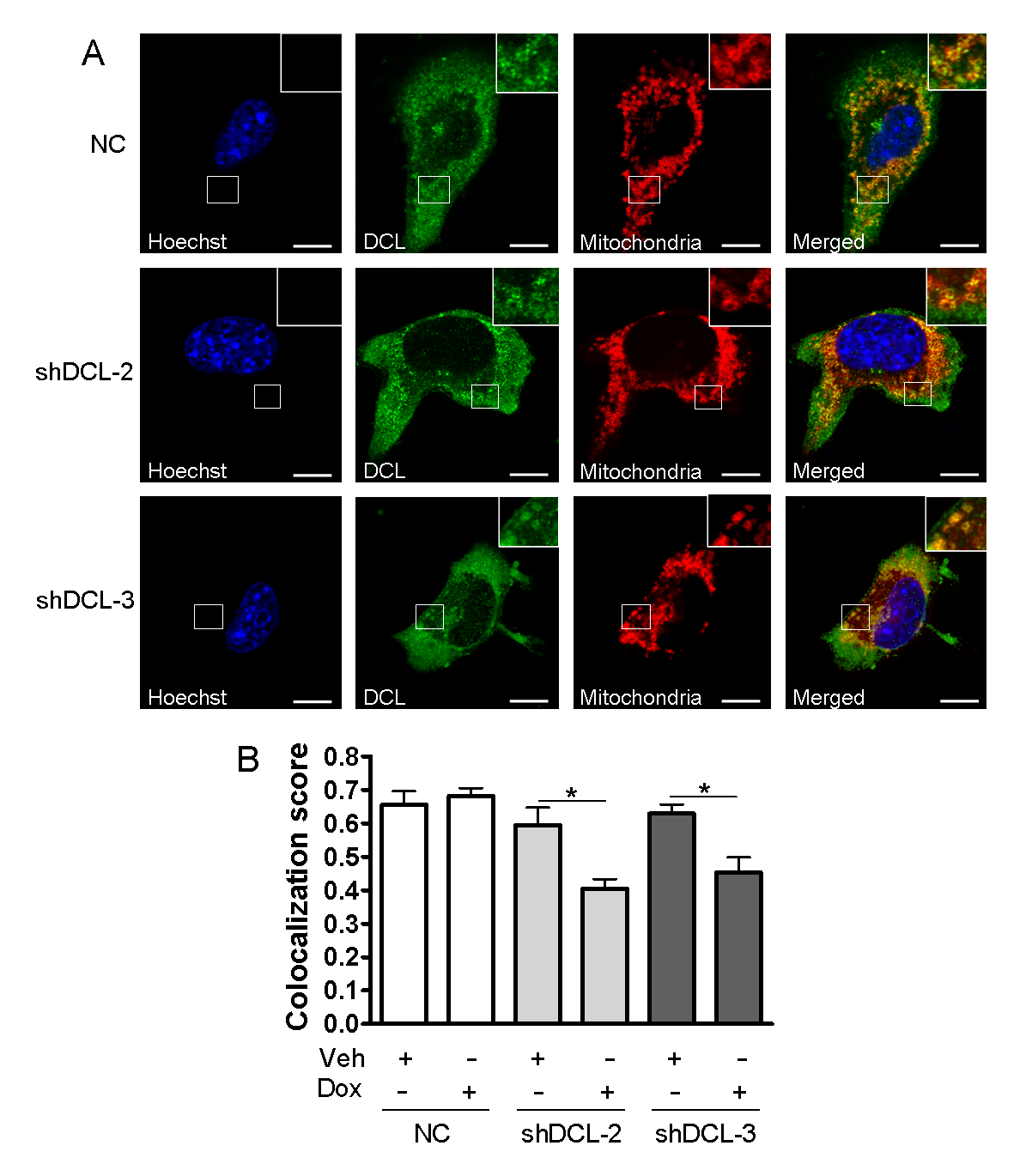

Supplement: Figure S4 — DCL colocalizes with mitochondria. (A) DCL (green), mitochondria (red) and nuclei (Hoechst, blue) staining in Dox-inducible NB cells treated with vehicle (Veh). (B) Colocalization scores in Dox-inducible NB cells after 72 hours doxycycline (Dox)- or Veh-treatment. Colocalization score was quantified using ImageJ as describes previously (Fitzsimons et al., 2008). NC, negative control Dox-inducible NB cells. shDCL-2 and shDCL-3, Dox-inducible NB cell lines that express a shRNA against DCL. Scale bars, 10 µm. Error bars, S.E.M. *, P < 0.05. (TIF) [file pone.0075752.s004.tif]

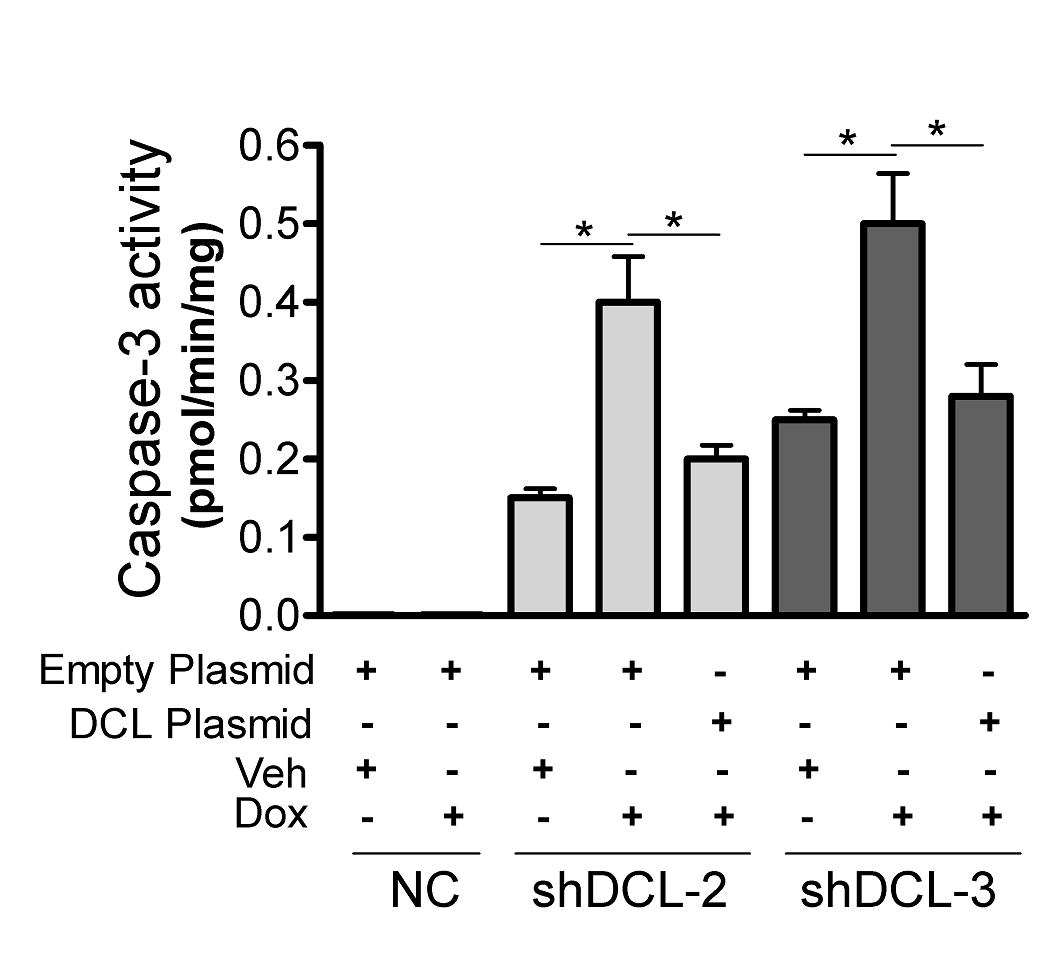

Supplement: Figure S5 — Recovery of DCL expression results in a decrease in caspase-3 activity. Caspase-3 activity in Dox-inducible NB cells (NC, shDCL-2 and shDCL-3) transfected with DCL or empty plasmid is shown. Transfection was performed 72 hours after starting doxycycline (Dox)- or vehicle (Veh)-treatment and caspase-3 activity was investigated 48 hours after the transfection. Error bars, S.E.M. *, P < 0.05. (TIF) [file pone.0075752.s005.tif]

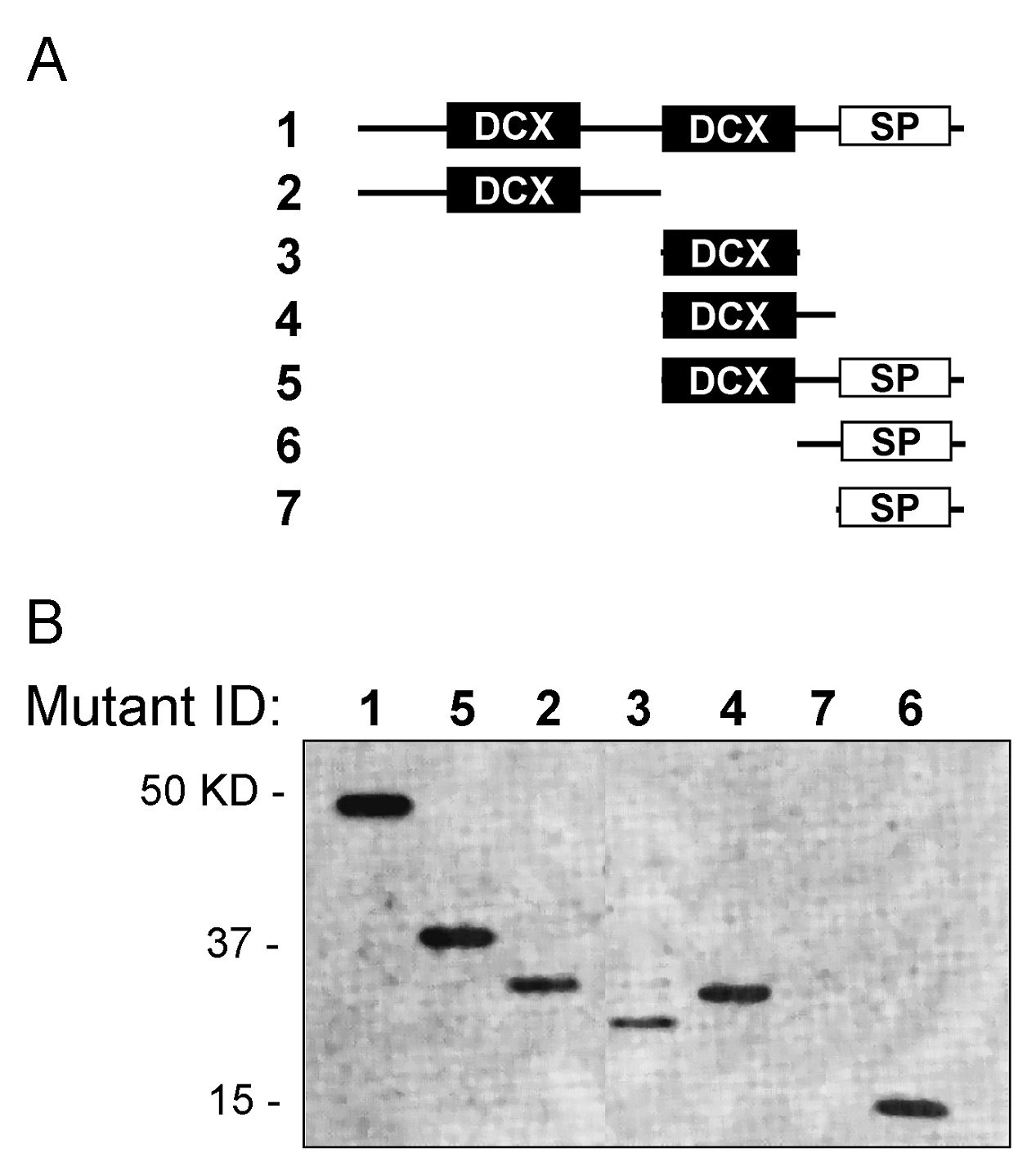

Supplement: Figure S6 — Expression of DCL and DCL mutants in COS-1 cells transfected with the different DCL sequences subcloned in pDsRed2-N1 vector. (A) Schematic representation of DCL full-length (1) and the different DCL mutants (2-7), showing the doublecortin domains (DCX-domains) and Serine/Proline (S/P)-rich domain (SP). (B) Western blotting results showing the expression of the fusion proteins DCL full-length-DsRed2 and DCL truncations-DsRed2. The expression of S/P-rich domain alone was not detectable, as previously reported (Vreugdenhil et al. 2007). (TIF) [file pone.0075752.s006.tif]

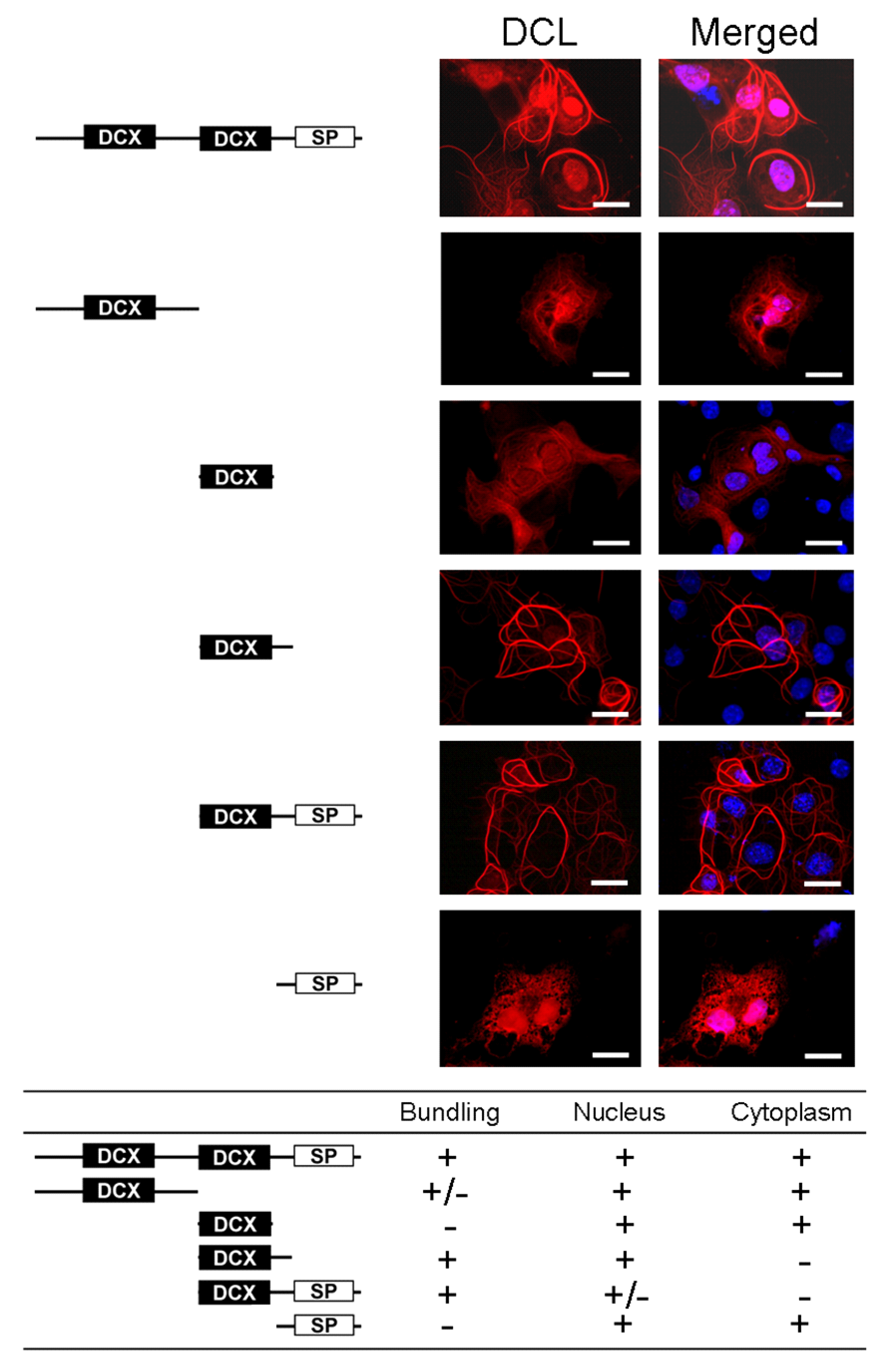

Supplement: Figure S7 — DCL full-length induces microtubule bundling and is located in the nucleus and cytoplasm. Immunofluorescence and colocalization of DCL full-length or DCL mutants (red) and nucleus (Hoechst staining, blue) in transfected COS-1 cells with different DCL sequences subcloned into pDsRed2-N1 vector. DCL full-length and the different DCL truncations were found present (+) or absent (-) in the nucleus, cytoplasm and/or in microtubule bundling. The second doublecortin (DCX) domain and the linker between this domain and Serine/Proline (S/P)-rich domain (SP) were found to be needed for microtubule bundling. +/-, present in few cells. Scale bars, 40 µm. (TIF) [file pone.0075752.s007.tif]
