# Supplementary material for: Silencing of Doublecortin-Like (DCL) Results in Decreased Mitochondrial Activity and Delayed Neuroblastoma Tumor Growth
Source: PLoS One. 2013 Sep 26;8(9):e75752. doi: 10.1371/journal.pone.0075752 (PMC3784435; doi:10.1371/journal.pone.0075752)
Supplement: Table S1 — (PDF) [file pone.0075752.s008.pdf]

Table S1. List of primers used to generate the different DCL truncations (Material and methods section)

	Primer sequences	
	Forward	Reverse
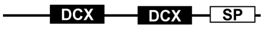	5’-ctcgagcaaccaatgtcgttcggcaga-3’	5’-cggtggatcccgcactgagtctcctga-3’
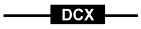	5’-ctcgagcaaccaatgtcgttcggcaga-3’	5’-cggtggatcccgcactgcccgggaggc-3’
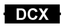	5’-ctcgagcaaccaatggcctcccgggcagtg-3’	5’-cggtggatcccgtgatgctattttggt-3’
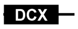	5’-ctcgagcaaccaatggcctcccgggcagtg-3’	5’-cggtggatcccggctggtggaggctgg-3’
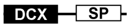	5’-ctcgagcaaccaatggcctcccgggcagtg-3’	5’-cggtggatcccgcactgagtctcctga-3’
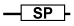	5’-ctcgagcaaccaatggcgtcccgcaga-3’	5’-cggtggatcccgcactgagtctcctga-3’
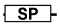	5’-ctcgagcaaccaatgtcagttaatgga-3’	5’-cggtggatcccgcactgagtctcctga-3’
