# Supplementary material for: Silencing of Doublecortin-Like (DCL) Results in Decreased Mitochondrial Activity and Delayed Neuroblastoma Tumor Growth
Source: PLoS One. 2013 Sep 26;8(9):e75752. doi: 10.1371/journal.pone.0075752 (PMC3784435; doi:10.1371/journal.pone.0075752)
Supplement: Table S2 — (PDF) [file pone.0075752.s009.pdf]

Table S2. Prediction of subcellular localization of the different DCL mutants based on the amino acid sequence (PSORT II)

	Subcellular localization
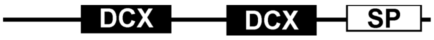	73.9 % nuclear; 13.0 % cytoplasmic; 13.0 % mitochondrial
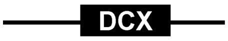	73.9 % nuclear; 17.4 % cytoplasmic; 8.7 % mitochondrial
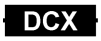	34.8 % cytoplasmic; 21.7 % mitochondrial; 21.7 % nuclear; 8.7 % vacuolar; 4.3 % cytoskeletal; 4.3 % peroxisomal; 4.3 % endoplasmic reticulum
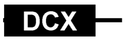	65.2 % nuclear; 30.4 % mitochondrial; 4.3 % cytoskeletal
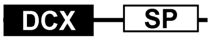	65.2 % nuclear; 30.4 % mitochondrial; 4.3 % cytoskeletal
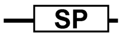	65.2 % mitochondrial; 17.4 % nuclear; 13.0 % cytoplasmic; 4.3 % endoplasmic reticulum
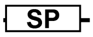	43.5 % mitochondrial; 30.4 % nuclear; 17.4 % cytoplasmic; 4.3 % cytoskeletal; 4.3 % peroxisomal
